# Controlling the Flexibility of MIL‐88A(Sc) Through Synthetic Optimisation and Postsynthetic Halogenation

**DOI:** 10.1002/chem.202201364

**Published:** 2022-06-29

**Authors:** Catherine A. Walshe, Alexander J. R. Thom, Claire Wilson, Sanliang Ling, Ross S. Forgan

**Affiliations:** ^1^ WestCHEM School of Chemistry University of Glasgow Joseph Black Building, University Avenue Glasgow G12 8QQ UK; ^2^ Advanced Materials Research Group, Faculty of Engineering University of Nottingham University Park Nottingham NG7 2RD UK

**Keywords:** breathing, chemisorption, flexible, metal-organic frameworks, postsynthetic modification

## Abstract

Breathing behaviour in metal‐organic frameworks (MOFs), the distinctive transformation between a porous phase and a less (or non) porous phase, often controls the uptake of guest molecules, endowing flexible MOFs with highly selective gas adsorptive properties. In highly flexible topologies, breathing can be tuned by linker modification, which is typically achieved pre‐synthetically using functionalised linkers. Herein, it was shown that MIL‐88A(Sc) exhibits the characteristic flexibility of its topology, which can be tuned by 1) modifying synthetic conditions to yield a formate‐buttressed analogue that is rigid and porous; and 2) postsynthetic bromination across the alkene functionality of the fumarate ligand, generating a product that is rigid but non‐porous. In addition to providing different methodologies for tuning the flexibility and breathing behaviour of this archetypal MOF, it was shown that bromination of the formate‐bridged analogue results in an identical material, representing a rare example of two different MOFs being postsynthetically converted to the same end product.

## Graphical abstract

**The flexibility of the MOF** MIL‐88A(Sc) is described and compared to its well‐established Fe analogue. Its breathing behaviour can be controlled through installation of formate bridges during synthesis that ensure rigidity by structural buttressing, or by postsynthetic bromination of the fumarate linker to induce rigidity by the steric influence of the bulky linker substituents.

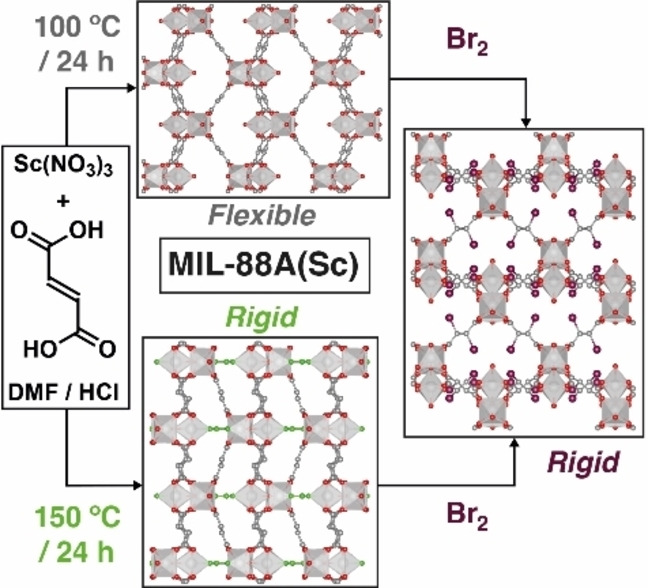

## Introduction

**Figure 1:**
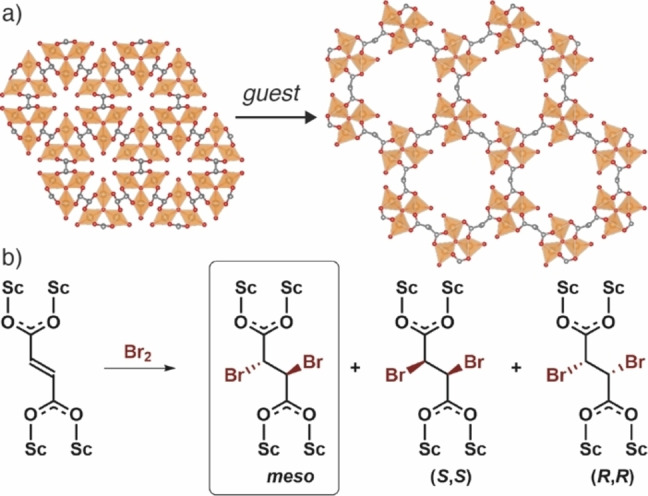
a) Expansion of MIL‐88A(Fe) from the dried to hydrated form visualised down the crystallographic *c* axis. Structure models redrawn from Ref. [9]; Fe, orange polyhedra; C, grey; O, red; H atoms removed for clarity. Not to scale. b) Schematic of postsynthetic bromination of the fumarate linker in MIL‐88A(Sc) showing the potential products of which the *meso* compound would be expected.

Metal‐organic frameworks (MOFs) have attracted increasing attention in recent years due to their versatile and highly tuneable structures, comprising metal‐containing secondary building units (SBUs) linked by organic ligands.^[1]^ This effectively infinite set of possible combinations yields multi‐dimensional extended frameworks that can be tailored to particular applications, notably gas storage^[2]^ and separation,^[3]^ catalysis^[4]^ and drug delivery.^[5]^ MOFs constructed using trivalent metal cations, in particular Al^3+^, Fe^3+^ and Cr^3+^, have been widely studied owing to the inherent flexibility that many of these frameworks exhibit.^[6]^ When combined with linear dicarboxylate linkers, they often adopt MIL‐88‐type structures (MIL=Materiaux de l'Institut Lavoisier), whereby the material constitutes trimeric [M_3_O(RCO_2_)_6_(H_2_O)_2_(X)] (M=metal, X=monoanion) SBUs bridged by dicarboxylate linkers into the **acs** topology with hexagonal channels running along the crystallographic *c* axis.^[7]^ This isoreticular class of materials has displayed remarkable breathing properties, swelling or contracting in response to external stimuli such as solvent immersion, pressure and temperature.^[8]^ For example, Férey et al. reported that MIL‐88A(Fe), an iron MOF constructed using fumarate linkers with formula [Fe_3_O(C_4_H_2_O_4_)_3_(H_2_O)_2_(X)], exhibits an 85 % increase in the unit cell volume between the dried, contracted form and the open, hydrated structure (Figure 1a).^[9]^ Other frameworks in this isoreticular series also exhibit these dynamic properties, for example MIL‐88D(Cr), where the linker is biphenyl‐4,4’‐dicarboxylate (bpdc), shows a 235 % increase in unit cell volume when the dried solid is immersed in pyridine.^[10]^

The swelling capacity in these systems is primarily a function of linker length, however, this is not the only method of controlling framework flexibility; some attempts to control the breathing behaviour have focussed on linker substitution.^[11]^ Horcajada et al. demonstrated that when the bpdc linker in MIL‐88D(Fe) is functionalised with bulky substituents such as methyl groups, the extent of breathing is reduced, as a result of the steric hindrance of the substituents reducing favourable π‐π interactions between linkers as the structure closes.^[8b]^ Similarly, for MIL‐88B(Fe), where the linker is benzene‐1,4‐dicarboxylate, di‐ or tetra‐substitution with bulky groups imparts permanent porosity, as the MOF cannot fully close to a non‐porous form.^[8b]^ This linker functionalization strategy has also allowed control over breathing and flexibility in a series of pillared Zn MOFs.^[12]^

An alternative to the direct use of functionalised linkers to form MOFs is postsynthetic modification (PSM), involving a chemical change to the framework while maintaining the overall framework crystallinity.^[13]^ Covalent transformations of pendant groups of the organic linkers are common; this often has an effect on gas sorption properties and occasionally, flexibility of the framework.^[14]^ For example, Carrington et al. have recently reported the PSM of SHF‐61 (SHF=Sheffield Framework), an interpenetrated In MOF which breathes in two dimensions along the crystallographic *b* and *c* axes, to yield an acetamide‐modified MOF, SHF‐62. The additional functionality on the pendant group of SHF‐62 induces breathing in a third dimension, due to unfavourable interactions between amide substituents.^[15]^

In recent years, increasing attention has been paid to the PSM of integral units of MOF linkers. In 2009, Bauer et al. demonstrated the diastereoselective bromination of stilbene‐4,4’‐dicarboxylate (SDC) linkers in a Zn MOF, however, the reaction required high temperatures, which resulted in degradation and a loss of porosity.^[16]^ Marshall et al. later reported the halogenation of a range of chemically and mechanically stable Zr and Hf MOFs with ligands containing unsaturated units such as alkenes and alkynes, and successfully monitored their single‐crystal to single‐crystal transformations by single crystal X‐ray diffraction.^[17]^ As well as in‐depth characterisation of bulk microcrystalline samples, they reported that the brominated MOFs displayed a decrease in the average elastic modulus compared to the parent structures, proposing that the resultant change in hybridisation of the central carbon atoms of the linkers increases the degrees of freedom.^[17b]^ Another group reported a reversible crystalline‐amorphous transformation of a Zr MOF containing the SDC linker upon bromination of the internal alkene unit, and also related this to increased linker motion.^[18]^

In these cases, PSM of integral unsaturated C−C bonds in the linkers has increased flexibility of the MOFs due to linker conformational change, but the underlying topologies do not breathe. To study the effect of integral PSM on a topologically flexible MOF, we selected MIL‐88A, specifically the Sc(III) congener. Sc MOFs typically show good chemical stability, which is required to facilitate PSM, while the fumarate linker of MIL‐88A(Sc) contains an accessible C=C double bond for halogenation (Figure 1b). In addition, we have previously shown Sc MOFs to be highly amenable to modulated self‐assembly, which was expected to allow access to single crystals suitable for X‐ray diffraction analysis.^[19]^ Herein we report the use of modulated self‐assembly to synthesise MIL‐88A(Sc), which we term **1**, and a related, formate‐bridged analogue, **2**. Careful characterisation of both MOFs shows that **1** exhibits the characteristic flexibility of the MIL‐88 series of MOFs, whilst **2** is rigid and permanently porous. Both MOFs can be successfully postsynthetically brominated to yield an identical final product, which further alters both flexibility and porosity.

## Results and Discussion

### Synthesis of 1

**Figure 2:**
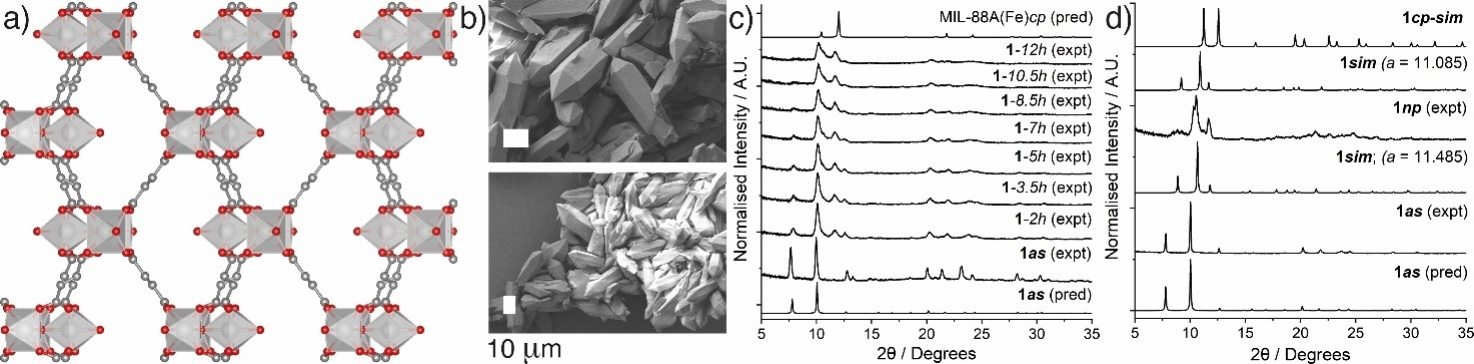
a) Crystal structure of **1** ***as*** viewed down the *a* axis; Sc, silver polyhedra; C, grey; O, red; H atoms removed for clarity. b) SEM images of **1** ***as*** showing characteristic hexagonal morphology. Scale bar 10 μm in both. c) Stacked powder X‐ray diffractograms of **1** ***as*** as it dries over time in air. d) Stacked powder X‐ray diffractograms comparing DFT models to experimental data to qualitatively assess the openness of **1** ***np***, a sample solvent exchanged in MeCN and dried under vacuum.

Initial solvothermal syntheses of highly crystalline samples of **1** were completed using scandium nitrate and fumaric acid in *N,N‐*dimethylformamide (DMF), with ten equivalents of hydrochloric acid (HCl) as a modulator (Supporting Information, Section S2). Highly crystalline powders and single crystals of **1** were reliably produced under these conditions at 100 °C for 24 h; the single crystal structure of **1** (Figure 2a), with formula [Sc_3_O(C_4_H_2_O_4_)_3_(H_2_O)_2_(OH)], was consistent with that reported by Wang et al. during the course of our study, from crystals prepared under different reaction conditions.^[20]^ Once cooled to room temperature, the materials were washed with fresh DMF three times before powder X‐ray diffraction (PXRD) measurements were collected, and further solvent exchange was carried out where mentioned. The PXRD pattern collected of a sample in DMF, herein known as **1** ***as*** (as‐synthesised), matches closely with the calculated pattern from our single crystal structure, indicating the phase‐purity of the sample. SEM imaging provides further evidence of the identity of the sample (Figure 2b) where **1** ***as*** has a hexagonal rod‐like morphology, consistent with reported MIL‐88‐type frameworks.^[21]^

The flexibility of the iron analogue MIL‐88A(Fe) is well‐reported,^[9]^ but was not detailed in the recent report of MIL‐88A(Sc),^[20]^ and so, initially, attempts were made to collect single crystal structures of samples of **1** ***as*** that were allowed to slowly dry in air (Supporting Information, Section S3). Unfortunately, crystal degradation meant only two reliable sets of unit cell parameters could be collected, which showed small but appreciable contractions in unit cell volume on loss of DMF solvent (*V*=1700(3) and 1840.6(7) Å^3^, respectively) compared to **1** ***as*** (*V*=2073.8(11) Å^3^), while maintaining the P6_3_/m space group of the parent MOF. Comparison to the previously reported closed structure of MIL‐88A(Fe), which has *V*=1135 Å^3^,^[9]^ confirms that neither sample is fully closed. PXRD measurements were therefore carried out on bulk samples of **1** ***as*** whilst they dried directly from DMF at room temperature in air (Figure 2c). **1** ***as*** again contracts as residual DMF diffuses out of the pores, leading to a gradual shift of the Bragg peaks to higher 2θ angles with concomitant broadening that makes indexing the unit cell difficult. This is suggestive of a continuous change in composition across multiple closely related phases as the sample closes. Further contraction and similar broadening of Bragg peaks are observed when **1** ***as*** is dried under vacuum from DMF. Whilst it was not possible to determine the unit cell parameters of these dried samples, comparison of diffractograms with those of MIL‐88A(Fe)^[9]^ again indicate that the sample is far from fully closed.

Solvent exchange was therefore utilised to attempt to access a fully closed sample of **1**. Samples of **1** ***as*** were soaked in different solvents for 7 days and were exchanged with fresh solvent, before being dried overnight in a vacuum desiccator and subsequently analysed by PXRD (Supporting Information, Section S4). The dynamic behaviour of **1** is evident in PXRD patterns collected following solvation and subsequent evacuation, whereby the framework contracts to a relatively similar extent across the solvent range that was studied, but with notable exceptions. The diffractograms again show broad Bragg reflections suggesting anisotropic breathing and making indexing difficult. Density functional theory (DFT) calculations were therefore utilised to attempt to interpret the diffraction data, and a closed structural model of **1**, termed **1** ***cp‐sim***, was generated (Supporting Information, Section S4). First, the open‐pore structure of **1** was computationally optimised by DFT, and then further optimised under pressure, which forced the structure to undergo the open to closed phase transformation. Subsequently, the now closed‐pore structure was re‐optimised under ambient pressure to obtain the lattice parameters of the closed‐pore structure under ambient pressure, **1** ***cp‐sim***. The unit cell volume (*V*=1123 Å^3^) is very close to that previously determined for MIL‐88A(Fe) (*V*=1135 Å^3^), showing its validity.^[9]^ To interpret the experimental powder‐X‐ray diffraction data, a range of structures of **1** with different unit cell volumes (i.e. at different levels of closing) was generated. Starting from the DFT‐optimised structure **1** ***cp‐sim***, a series of *a/b* axes parameters from 9.485 Å to 13.485 Å was selected. Partial unit cell optimisations, i.e., only allowing the *c* axis parameter to relax while keeping the *a/b* cell parameters fixed during the optimisation, were performed. From this, a series of structural models of **1**, named **1** ***sim*** (*a*=x) where x=the fixed *a* axis parameter, with unit cell volumes ranging from 1213 Å^3^ to 2126 Å^3^, was generated and used to predict powder X‐ray diffractograms as **1** closes. Qualitative comparison of powder X‐ray diffractograms (Figure 2d) suggest that samples of **1** ***as*** dried from both EtOH and MeCN form the most closed structures, with unit cell volumes of approximately 1600 Å^3^ (a tentative Pawley refinement for a sample dried from EtOH gives a unit cell volume of 1618 Å^3^, Supporting Information Figure S6) which are at intermediate levels of closure compared to **1** ***cp‐sim***. ^1^H NMR spectroscopic analysis of digested samples (Supporting Information, Figure S7) shows the attempted solvent exchanges do not successfully remove all residual DMF from the pores of **1**, which we believe results in intermediate paracrystalline phases being accessed.

**Figure 3:**
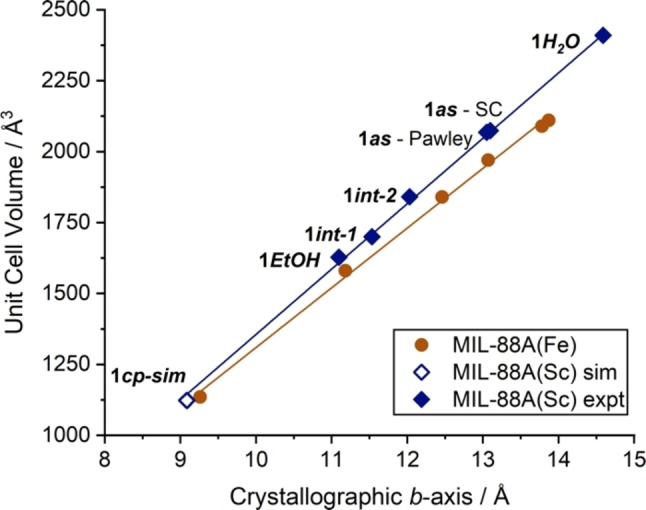
Plot of unit cell volume vs. crystallographic *b* axis for varying datasets collected for MIL‐88A(Sc) (**1**) compared to data previously published for MIL‐88A(Fe) from Ref. [9]. Data are tabulated in the Supporting Information, Table S2.

To further assess flexibility, it was attempted to swell **1** ***as*** by solvent exchange with water, which had previously produced the most open form of MIL‐88A(Fe) (*V*=2110 Å^3^).^[9]^ PXRD analysis of a sample of **1** ***as*** soaked in water at room temperature for one hour showed significant swelling, with a Pawley refinement giving a unit cell volume of 2410(16) Å^3^ (Supporting Information, Figure S13). This suggests that, should a closed pore sample be accessible, **1** has a potentially greater amplitude of flexibility than MIL‐88A(Fe), likely due to the increased ionic radius of Sc^3+^ compared to Fe^3+^. Relationships between the lengths of the crystallographic axes and unit cell volume for samples of **1** have been plotted against those previously reported for solvated MIL‐88A(Fe) samples,^[9]^ further demonstrating the effect of ionic radius on the breathing behaviours of the two isostructural MOFs (Figure 3).

After screening several solvents of varying polarities and sizes, acetonitrile‐exchanged **1** ***as*** was taken forward, as MeCN reliably yields a narrow‐pore material which has partially closed, and MeCN was previously used as a solvent of choice in solution‐based postsynthetic halogenation of MOFs.^[17c]^ This material is herein known as **1** ***np*** (np=narrow pore) on which all further analyses will be based (Supporting Information, Section S5). Following further activation under vacuum at 150 °C for 20 h, ^1^H NMR spectroscopic analysis of digested samples indicates 1.5 moles of DMF per Sc_3_O SBU remain in the pores of **1** ***np***, and likely explains why a fully closed structure has not been achieved under these conditions. The presence of residual DMF is further confirmed using elemental analysis, where the measured nitrogen content of 3.23 % wt correlates well with the value calculated (3.20 % wt) from the formula [Sc_3_O(C_4_H_2_O_4_)_3_(H_2_O)_2_(OH)]⋅(C_3_H_7_NO)_1.5_. TGA measurements were carried out in air, to understand the thermal stability of **1** and further probe the solvent content. **1** exhibited a high thermal stability, with degradation of the framework occurring at 460 °C, resulting in a residual mass of 31.0 % wt when normalised to remove contribution from adsorbed water below 100 °C. This is concordant with an expected Sc_2_O_3_ residue of 31.5 % for the above formula predicted by elemental analysis and NMR spectroscopy.

The characteristic anisotropic breathing behaviour in MIL‐88‐type frameworks often underpins their investigation for gas capture/separation applications.^[22]^ However, a combination of the contraction of **1** and the residual DMF in the pores results in limited nitrogen adsorption at 77 K; following activation at 150 °C under vacuum for 20 h, there is minimal N_2_ uptake by **1** ***np***. In contrast, **1** ***np*** displays reasonable CO_2_ adsorption −2.4 mmol g^−1^ at 273 K and 1.7 mmol g^−1^ 298 K, both at 1 bar− which is comparable with other reported Sc MOFs.^[23]^ In the absence of polar functional groups in a MOF, reports have indicated that CO_2_ uptake can be improved by tuning pore size using smaller linkers.^[24]^ **1** ***np*** also achieves a modest H_2_ adsorption of 1.3 wt%, which is relatively unremarkable when compared with other reported Sc‐MOFs.[^8d^, ^25^]

### Synthesis of 2

**Figure 4:**
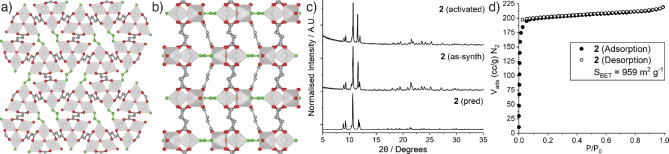
Crystal structure of **2** as viewed down a) the crystallographic *b* axis and b) crystallographic *c* axis; Sc, silver polyhedra; C, grey; O, red; formate bridges, green; H atoms and pore solvents removed for clarity. c) Stacked powder X‐ray diffractograms of **2** as‐synthesised, and after activation at 150 °C under vacuum, compared to that predicted from the crystal structure, showing its rigidity. d) N_2_ adsorption/desorption isotherms (77 K) of an activated sample of **2**.

During synthetic optimisations, a new Sc‐fumarate MOF named **2**, with an orthorhombic unit cell (*a=*17.0967(9), *b=*15.1239(9), *c=*12.2795(7) Å), was discovered at higher synthesis temperatures (150 °C) with scandium nitrate and fumaric acid, again in DMF but with one equivalent of HCl as modulator (Supporting Information, Section S6). The framework has the same trigonal prismatic SBU as **1**, where three scandium centres are linked by one *μ*_3_‐O^2−^ centre and capped by carboxylate groups from the fumaric acid linkers, with the same underlying connectivity (Figures 4a, 4b). However, each cluster is now bridged to two others by formate anions coordinated to axial Sc positions, which replace the terminal OH anions and one of the two coordinating water ligands of **1**. Only one of the three Sc^3+^ ions in the SBU has axially coordinated solvent a 1 : 1 ratio of occupationally disordered water and *O*‐coordinated DMF (itself disordered over two positions) with each water ligand hydrogen bonding to a further pore‐bound DMF molecule to yield a material with overall formula [Sc_3_O(C_4_H_2_O_4_)_3_(HCOO)(H_2_O)_0.5_(C_3_H_7_NO)_0.5_] ⋅ (C_3_H_7_NO)_0.5_. This formate bridging is similar to that observed by Wei et al. in rare‐earth MOFs with underlying MIL‐88 topologies, whereby the trinuclear SBUs are interconnected by HCOO^−^ groups to enhance chemical and thermal stability,^[26]^ but these examples, with benzene‐1,4‐dicarboxylate and naphthalene‐2,6‐dicarboxylate ligands, differ in overall connectivity and coordination compared to **2**. Most recently, formate bridged Sc_3_O trimers have been reported by Prasad et al. when synthesising Sc‐BTB (BTB=benzenetribenzoate, or 1,3,5‐tris(4‐carboxyphenyl)benzene) resulting in a chain‐like SBU, rather than independent trimers.^[23a]^ They reported that the formate groups are a direct result of the hydrolysis of DMF at higher temperatures in the presence of acid, similar to the synthetic conditions employed to prepare **2**.

Following synthesis, **2** was washed in fresh DMF three times, and subsequently by fresh acetonitrile three times, and dried under vacuum overnight before further analysis. The PXRD pattern for a bulk sample of this as‐synthesised **2** closely matches the pattern predicted from the single crystal structure (Figure 4c). The ^1^H NMR spectrum of a digested sample confirms the presence of formate and DMF in expected quantities, while elemental analysis and TGA further support the bulk composition matching that of the crystal structure (Supporting Information, Section S6). The TGA profile reveals that **2** is thermally stable until approximately 370 °C, where it exhibits a large mass loss relating to the decomposition of the framework. This is similar to the thermal activity exhibited by **1**, however, the cleavage of the formate bridges induces a faster breakdown of the MOF at lower temperatures. As previously discussed, MOFs in the MIL‐88 series are well‐known for their flexible behaviour, however, following evacuation under vacuum at 150 °C, no changes are apparent in the powder X‐ray diffractogram of **2**, suggesting the formate bridges buttress the structure and do not allow it to close (Figure 4c). As such, **2** exhibits a type I isotherm for N_2_ adsorption at 77 K, with a relatively high BET area (*S*_BET_ = 959 m^2^ g^−1^, Figure 4d), and also displays good CO_2_ adsorption −2.7 mmol g^−1^ at 273 K and 1.9 mmol g^−1^ 298 K, both at 1 bar− values slightly higher than **1** ***np*** and comparable to other scandium MOFs^[23]^ but lower than the recently reported MIL‐142(Sc)‐PTB‐NH_2_ (Supporting Information, Figure S25). **2** also shows comparable H_2_ adsorption to **1** ***np,*** achieving an uptake of 1.36 wt%. Incorporation of the formate bridges has imparted rigidity to **2**, preventing the framework from flexing and closing, therefore eradicating the dynamic behaviour exhibited by **1**.

### Synthesis of 1‐Br_2_

**Figure 5:**
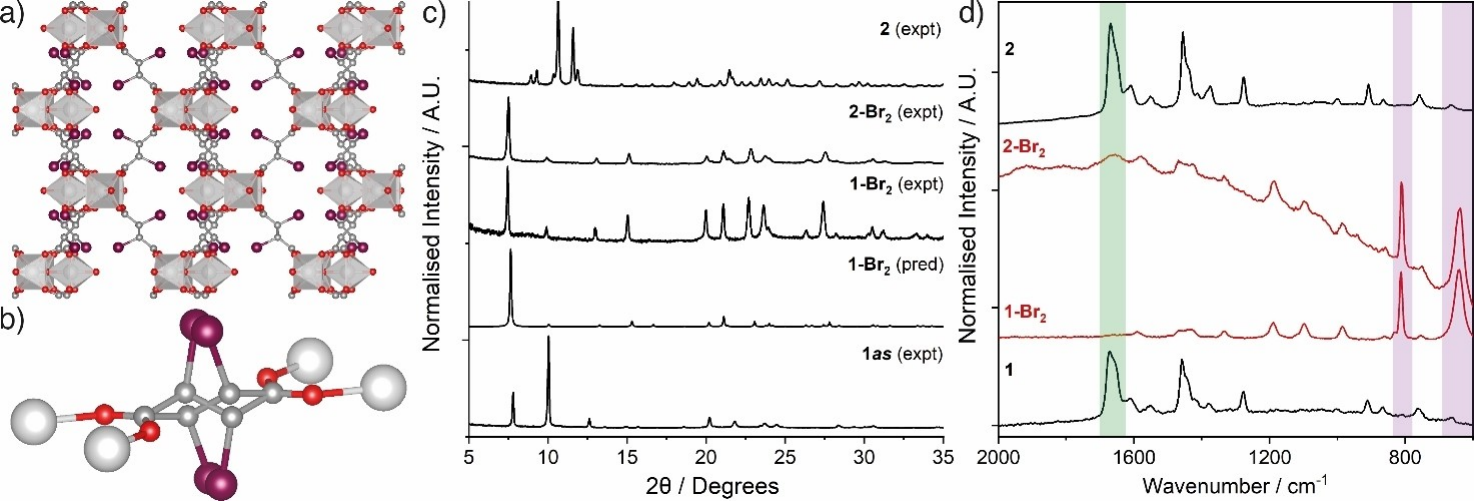
a) Crystal structure of **1‐Br_2_** viewed down the crystallographic *a* axis; Sc, silver polyhedra; C, grey; O, red; Br, purple; disorder and H atoms removed for clarity. b) Disorder in the brominated linker in the crystal structure of **1‐Br_2_**; Sc represented as silver spheres; H atoms removed for clarity. c) Stacked powder X‐ray diffractograms, and d) stacked Raman spectra showing conversion of **1** and **2** into identical brominated products, **1‐Br_2_** and **2‐Br_2_**, respectively. The green box highlights the band assigned to the C=C bond in **1** and **2** around 1650 cm^−1^, while the purple boxes highlight the bands assigned to the C−C (∼800 cm^−1^) and C−Br (∼650 cm^−1^) bonds in 1–1**‐Br_2_** and **2‐Br_2_**. The broad feature in the spectrum of **2‐Br_2_** is associated with laser‐induced sample degradation.

With the flexible **1** and the rigid **2** in hand, we sought to examine the effect of postsynthetic bromination of the fumarate linkers on the flexibility of both MOFs. Postsynthetic bromination reactions are often carried out on suspensions of the MOF in organic solvents,[^16^, ^17^] however, here we report the use of direct vapour diffusion to induce bromination, which we had previously used for postsynthetic iodination.^[17c]^ While carrying out this work, a similar method was reported by Matemb Ma Ntep et al. to brominate acetylenedicarboxylate linkers in a Ce(IV) MOF.^[27]^ **1** ***np*** and bromine liquid were added to separate, uncapped vials that were sealed in a closed vessel and allowed to react at room temperature for 48 h in darkness (Supporting Information, Section S7). The resulting material, which we have termed **1‐Br_2_**, was subsequently washed in acetonitrile to remove excess bromine from the pores and dried at room temperature under vacuum for further analysis. Concurrently, single crystals of **1** ***as*** were suspended in acetonitrile and Br_2_ added directly; the resultant single‐crystal to single‐crystal transformation allowed the collection of the single crystal structure of **1‐Br_2_** (Figure 5a).

The single crystal structure of **1‐Br_2_** reveals that the brominated MOF retains the same hexagonal space group P6_3_/m as the parent MOF, **1** ***as***. The chemical transformation to **1‐Br_2_** results in a small contraction along the *c* axis, from 13.958(4) Å to 13.538(1) Å, and a subsequent minor increase in unit cell volume from 2073.8(11) Å^3^ to 2088.4(5) Å^3^. The bromine units are clearly visible, with two equally occupied linker conformations evident in the disorder model (Figure 5b). PXRD patterns of bulk samples prepared by vapour phase bromination also confirm that there is a clear transformation from **1** ***np*** to **1‐Br_2_**. The PXRD pattern for **1‐Br_2_** predicted from the crystal structure exhibits a close match to the experimental diffractogram (Figure 5c), and the structural transition is confirmed by Pawley refinement (Supporting Information, Figure S27), indicating the bulk sample has been successfully brominated with high phase purity.

The chemical transformation can also be monitored by Raman spectroscopy, where the alkene (C=C) band at 1670 cm^−1^ disappears upon full bromination of the structure, alongside appearance of a strong band at 641 cm^−1^ which is indicative of the C−Br stretch (Figure 5d). Quantitative conversion to **1‐Br_2_** is also evident through ^1^H and ^13^C NMR spectroscopy of acid‐digested (D_2_SO_4_/DMSO‐*d*_6_) samples. The disappearance of the resonance at δ=6.6 ppm, assigned to the alkene protons of fumarate, and the emergence of a singlet at δ=4.5 ppm in the ^1^H NMR spectrum of **1‐Br_2_** indicates that **1** ***np*** has been fully and stereoselectively brominated to the expected *meso* product.^[28]^ Similarly, in the ^13^C NMR spectrum of **1‐Br_2_**, the emergence of a peak at δ=42 ppm and disappearance of peak at δ=134 ppm indicates the change in hybridisation of the alkene carbon atom upon bromination, from *sp* ^2^ to *sp* ^3^ (see Supporting Information, Figures S29 and S30). The ^1^H NMR spectrum indicates that the pore bound DMF from **1** ***as*** has been retained through the bromination process, giving an overall formula of [Sc_3_O(C_4_H_2_O_4_Br_2_)_3_(H_2_O)_2_(OH)] ⋅ (C_3_H_7_NO)_1.5_. Chemical modification is also evident using TGA; when residual weakly‐bound solvents are removed, the Sc_2_O_3_ residue (17.5 % wt) is close to that expected for the above formula (18.2 % wt). Elemental analysis, however, gave C and N contents lower than would be expected, correlating more closely with only one DMF molecule per SBU and adsorption of one water molecule, suggesting a formula of [Sc_3_O(C_4_H_2_O_4_Br_2_)_3_(H_2_O)_2_(OH)] ⋅ C_3_H_7_NO ⋅ H_2_O for this particular sample; the theoretical bromine content from this composition (42.9 % wt) correlates well with the experimentally measured value of 45.7 % wt.

Taken together, these comprehensive structural, spectroscopic, and compositional experiments confirm quantitative bromination of **1** ***np***. Whilst **1** ***np*** does not adsorb N_2_, the fact that bromination occurs throughout the bulk of the material in the vapour phase experiment confirms the pores are accessible to the Br_2_ molecule, but not to N_2_. The kinetic diameter of Br_2_ has been reported to be smaller than for N_2_, but they are very similar sized.^[29]^ One hypothesis could be greater adsorbent‐adsorbate interactions opening the pores, or alternatively bromination occurs at the particle surface, beginning to open the pores and allowing Br_2_ to penetrate further into the material to react and open adjacent pores to Br_2_. It is important to note, however, that bromination occurs at room temperature while N_2_ adsorption is measured at 77 K; this low temperature may nullify dynamic behaviours which would otherwise allow small molecule adsorption.

**Figure 6:**
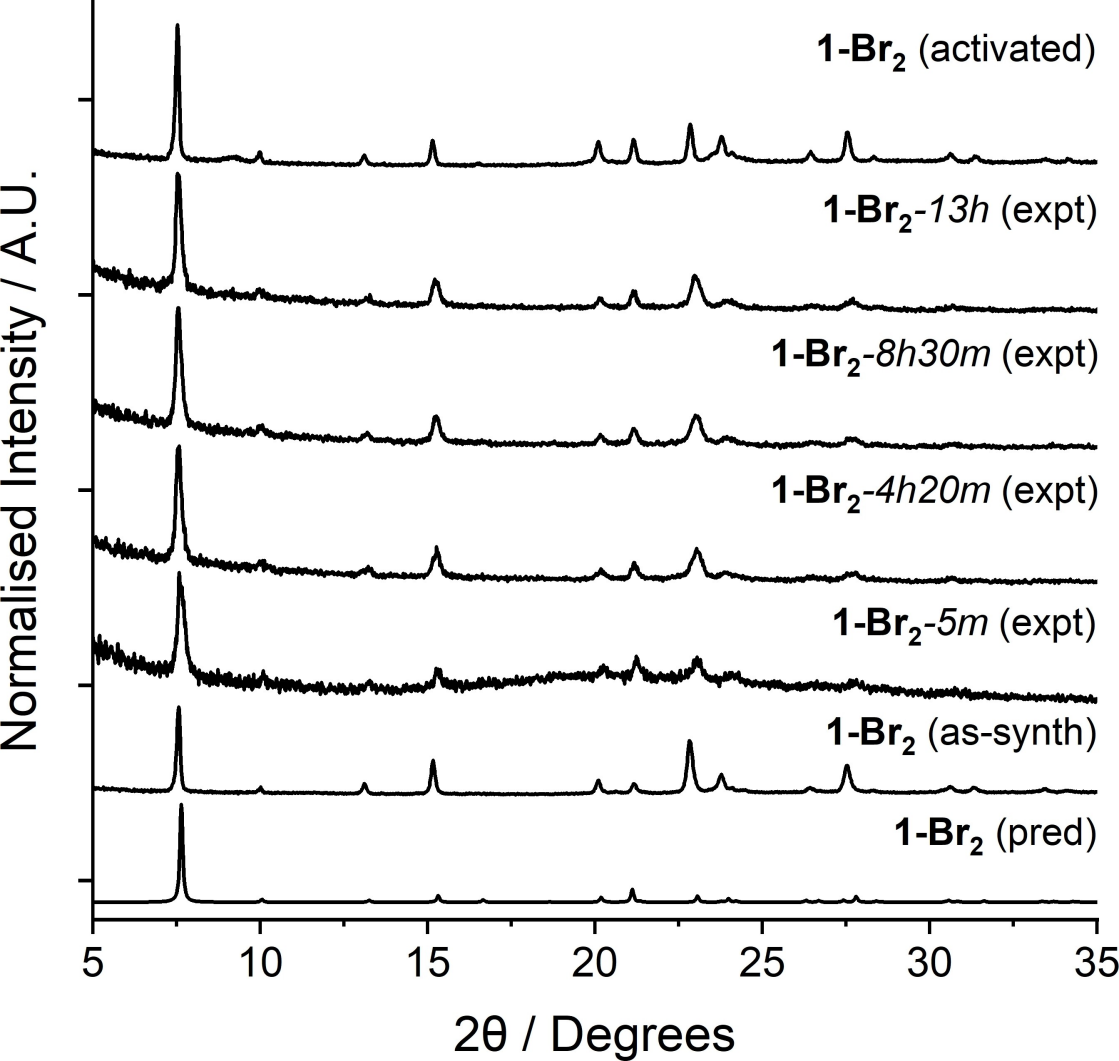
Stacked experimental powder X‐ray diffractograms of **1‐Br_2_** in the as‐synthesised and activated forms, compared with the diffractogram predicted from its crystal structure and a sample that was soaked in DMF and air dried (the time in the diffractogram label represents the drying time). All indicated no swelling/breathing behaviour on solvation and desolvation.

Whilst the addition of the bromine moieties across the unsaturated bond and subsequent change in hybridisation of the linker carbon atoms from *sp* ^2^ to *sp* ^3^ may be expected to increase flexibility, bromination of **1** actually imparts rigidity to the framework. Unlike **1** ***np***, **1‐Br_2_** shows no change in its powder X‐ray diffractogram when dried from, or soaked in, various solvents over an extended time. Even following activation for 20 h at 150 °C under vacuum, the framework shows no crystallographic changes according to PXRD (Figure 6). It is likely that the bulky bromine groups are sterically sufficient to prevent the framework from closing under ambient conditions. Despite the rigidity of **1‐Br_2_**, it would appear that the added bromine units sterically block the pores, and so N_2_ uptake at 77 K and 1 bar is negligible. Nevertheless, this PSM methodology yields a MOF with new chemical functionality and modified breathing behaviour, a change which, in this instance, could not have been achieved through solvent exchange alone.

Although both **1** and **2** are structurally independent, when **2** is subjected to the same bromination conditions it yields a material identical to **1‐Br_2_** (Supporting Information, Section S8). For ease of analysis and interpretation, this product will be herein called **2‐Br_2_**, and it displays an almost identical powder X‐ray diffractogram to **1‐Br_2_** (Figure 5c). The presence of the alkene bond in the fumaric acid linker remains the key target for reaction of the bromine vapours, however, the formate bridges are also unexpectedly lost. Both the chemisorption of Br_2_ across both the double bond of fumarate, and the loss of the formate bridges are evident in the ^1^H NMR spectrum of a digested sample, whereby the resonances at δ=6.6 and δ=8.1 ppm, assigned to the fumarate alkene protons and the formate protons, respectively, disappear following exposure to bromine, leaving a singlet at δ=4.5 ppm assigned to the now *sp* ^3^ hybridised linker C−H group (see Supporting Information, Figures S35 and S36). Bromination is also confirmed using Raman spectroscopy (Figure 5d). ^1^H NMR spectra indicated the retention of DMF – around 0.75 molecules per SBU compared to one molecule per SBU in the parent material **2** – while TGA is also commensurate with a formula of [Sc_3_O(C_4_H_2_O_4_Br_2_)_3_(H_2_O)_2_(OH)] ⋅ (C_3_H_7_NO)_0.75_ (observed Sc_2_O_3_ residue, 19.1 % wt; predicted Sc_2_O_3_ residue, 19.1 % wt). Elemental analysis again suggested adsorption of water prior to analysis, giving a formula for this sample of **2‐Br_2_** of [Sc_3_O(C_4_H_2_O_4_Br_2_)_3_(H_2_O)_2_(OH)] ⋅ (C_3_H_7_NO)_0.75_ ⋅ (H_2_O)_2_. The theoretical bromine content for this composition (43.0 % wt) correlated well with the experimentally measured value (43.5 % wt), which is indicative of bromine only reacting across the double bonds (expected: 43.0 %), rather than bromide also replacing the formate as a coordinating counterion on the SBU (expected: 47.4 %). The halogenation step has also impacted the gas sorption properties; **2** exhibits a BET area for N_2_ at 77 K of 959 m^2^ g^−1^ but, like **1‐Br_2_**, **2‐Br_2_** again shows negligible N_2_ uptake. The bromination of **1** and **2** therefore represents an unusual example whereby postsynthetic modification of two different MOFs results in the same final material.

## Conclusion

We have shown that the continuous breathing behaviour of MIL‐88A(Sc) (**1**) can be controlled both through direct synthetic methods as well as postsynthetic installation of linker functionality. By adjusting the reaction conditions for the modulated self‐assembly of **1**, a rigid and porous analogue, **2**, can be synthesised. Whilst **1** exhibits the flexibility characteristic of its topology and closes on removal of pore‐bound guests, **2** shows enhanced gas sorption properties as a consequence of bridging formate linkers buttressing the MOF enforcing structural rigidity. Quantitative postsynthetic bromination of the fumarate linkers in **1** and **2** yields, in each case, an identical product, which exhibits structural rigidity resulting from the installation of sterically bulky bromine substituents across the linkers. In addition to an unusual example of two different MOFs being postsynthetically modified to form a uniform product, we have identified MIL‐88A(Sc) as an efficient, irreversible adsorbent for Br_2_, as the dry MOF can theoretically chemisorb 0.88 g g^−1^ Br_2_ vapour. In particular, the study highlights the extent of pre‐and post‐synthetic control that can be exercised over MOF flexibility and gas adsorption properties, offering new design principles and the ability to carefully tune breathing in highly studied frameworks.

## Conflict of interest

The authors declare no conflict of interest.

1

## Supporting information

As a service to our authors and readers, this journal provides supporting information supplied by the authors. Such materials are peer reviewed and may be re‐organized for online delivery, but are not copy‐edited or typeset. Technical support issues arising from supporting information (other than missing files) should be addressed to the authors.

Supporting Information

## Data Availability

The data that support the findings of this study are openly available in University of Glasgow, Enlighten repository at https://doi.org/10.5525/gla.researchdata.1284, reference number 1284.
