# Supplementary material for: Controlling the Flexibility of MIL‐88A(Sc) Through Synthetic Optimisation and Postsynthetic Halogenation
Source: Chemistry. 2022 Jun 29;28(48):e202201364. doi: 10.1002/chem.202201364 (PMC9540238; doi:10.1002/chem.202201364)
Supplement: Supplementary file 1 — Supporting Information [file CHEM-28-0-s001.pdf]

Chemistry–A European Journal

Supporting Information

Controlling the Flexibility of MIL-88A(Sc) Through Synthetic Optimisation and Postsynthetic Halogenation

Catherine A. Walshe, Alexander J. R. Thom, Claire Wilson, Sanliang Ling, and Ross S. Forgan*

Supporting Information

Table of Contents

S1. General Experimental Remarks S2

S2. Synthesis of **1*as*** S4

S3. Flexibility of **1*as*** Under Drying S8

S4. Solvent Exchange of **1*as*** S10

S5. Characterisation of **1*np*** S24

S6. Synthesis and Characterisation of **2** S28

S7. Bromination of **1** S35

S8. Bromination of **2** S44

S9. References S52

S1. General Experimental Remarks

Unless otherwise stated, all reagents were purchased from Alfa Aesar, Tokyo Chemical Industry, Sigma Aldrich, Fluorochem or Strem, and used without any further purification.

Thermogravimetric analysis: TGA measurements were carried out in air using a TA Instruments Q500 Thermogravimetric Analyser with a heating rate of 10 °C min^{-1} and a heating range of 25 °C to 800 °C.

Powder X-Ray Diffraction: PXRD patterns were collected using a benchtop Rigaku MiniFlex 6G equipped with a D/teX Ultra detector, a 6-position (ASC-6) sample changer and Cu sealed tube ($K\alpha_1$ and $K\alpha_2$ wavelengths - 1.5406 and 1.5444 Å respectively) at 298 K. Scans ranged from 3-45° and were collected on spinning zero-background holders. All indexing and Pawley fitting was completed using GSAS-II software.[S1]

Nuclear Magnetic Resonance Spectroscopy: ^{1}H NMR and ^{13}C NMR spectroscopic data were recorded on a 400 MHz Bruker AVIII spectrometer. Digested MOFs were measured in DMSO-d_6/D_2SO_4 and all spectra were referenced to residual solvent peaks.

Single-Crystal X-Ray Diffraction: Measurements were carried out using Bruker D8 VENTURE diffractometer equipped with a Photon II CPAD detector, with an Oxford Cryosystems Helix device mounted on an IμS 3.0 (dual Cu and Mo) microfocus sealed tube generator. Data were collected at 150 K using Mo-Kα radiation (λ = 0.71073 Å) and structures were solved and refined using SHELXT[S2] and SHELXTL[S3] within the software Olex2.[S4]

Gas Sorption: Measurements were obtained on a Quantachrome Autosorb iQ machine. Samples were degassed at 150 °C for 20 hours prior to analysis, where nitrogen and hydrogen adsorption and desorption isotherms were collected at 77 K up to 1 bar. BET surface areas were calculated from the collected nitrogen isotherms using the Micropore BET Assistant in the Quantachrome AsiQwin software. Carbon dioxide adsorption isotherms were collected at 273 K, 288 K and 298 K up to 1 bar.

Raman Spectroscopy: Raman spectra were obtained using a Horiba Jobin-Yvon LabRAM HR800 system (532 nm Ar laser, 100 mW). An aperture size of 100 µm and a 10% filter was used in order to prevent sample degradation.

Bromine Analysis: Bromine analysis was carried out by MEDAC Ltd, Chobham, Surrey, GU24 8JB.

Elemental Analysis: CHN data were as collected using an Exeter CE440 Elemental Analyser, run in CHN mode with combustion temperature set to 955 °C and reduction temperature set to 650 °C.

Scanning Electron Microscopy: Samples were imaged using a Carl Zeiss Sigma variable Pressure Analytical SEM. Powders were deposited onto carbon tabs mounted on aluminium stubs and coated with Pd for 150 seconds using a Polaron SC7640 sputter coater.

S2. Synthesis of 1*as*

Synthesis of $Sc_3O(C_4H_2O_4)_3(H_2O)_2(OH)]\cdot nDMF$ (1*as*): Fumaric acid (0.04 g, 0.35 mmol) and scandium nitrate hydrate (0.10 g, 0.33 mmol) were added to a 25 mL Pyrex vial and *N,N*-dimethylformamide (DMF) (4 mL) was added. To this mixture, hydrochloric acid (HCl) (110 μL, 3.3 mmol) was added and the reaction mixture was sonicated for 10 minutes. The vial was placed in an isothermal oven at 100 °C for 24 hours. The vial was removed from the oven and allowed to cool to room temperature. Single crystals were collected by pipette from the mother solution and left to stand until further analysis. The bulk solid product was collected by centrifugation (4500 rpm, 10 minutes) and washed three times with fresh DMF. **1*as*** was left to stand in DMF until further analysis or activations were carried out, where mentioned.

Crystal data for 1*as*: $Sc_3O(C_4H_2O_4)_3(H_2O)_2(OH)$, M_r =546.09, crystal dimensions 0.16 x 0.06 x 0.02 mm, Hexagonal; $a = b$ = 13.098 (3) Å, c = 13.958 (4) Å, V= 2073.8 (11) Å^3, T = 150 K, space group $P6_3/m$ (No. 176), Z = 2, 12410 reflections measured, 1925 independent reflections (R_{int} = 0.146) which were all used in calculations. The final R_1 = 0.061 for 1026 observed data $R[F^2 > 2\sigma(F^2)]$ and $wR(F^2)$ = 0.192 (all data). CCDC deposition number 2150610.

Following the synthesis of **1*as***, it was washed with acetonitrile and dried at room temperature. A powder X-ray diffraction (PXRD) pattern was collected (Figure S1) which shows good agreement with the predicted pattern, generated from SCXRD data. This confirms the phase purity of **1*as***. A Pawley fit of the experimental PXRD pattern further confirms phase purity, yielding hexagonal unit cell parameters $a = b$ = 13.050(17), c = 14.022(2) Å, V = 2068(3) Å^3 (R_{wp} = 13.86%, Figure S2).

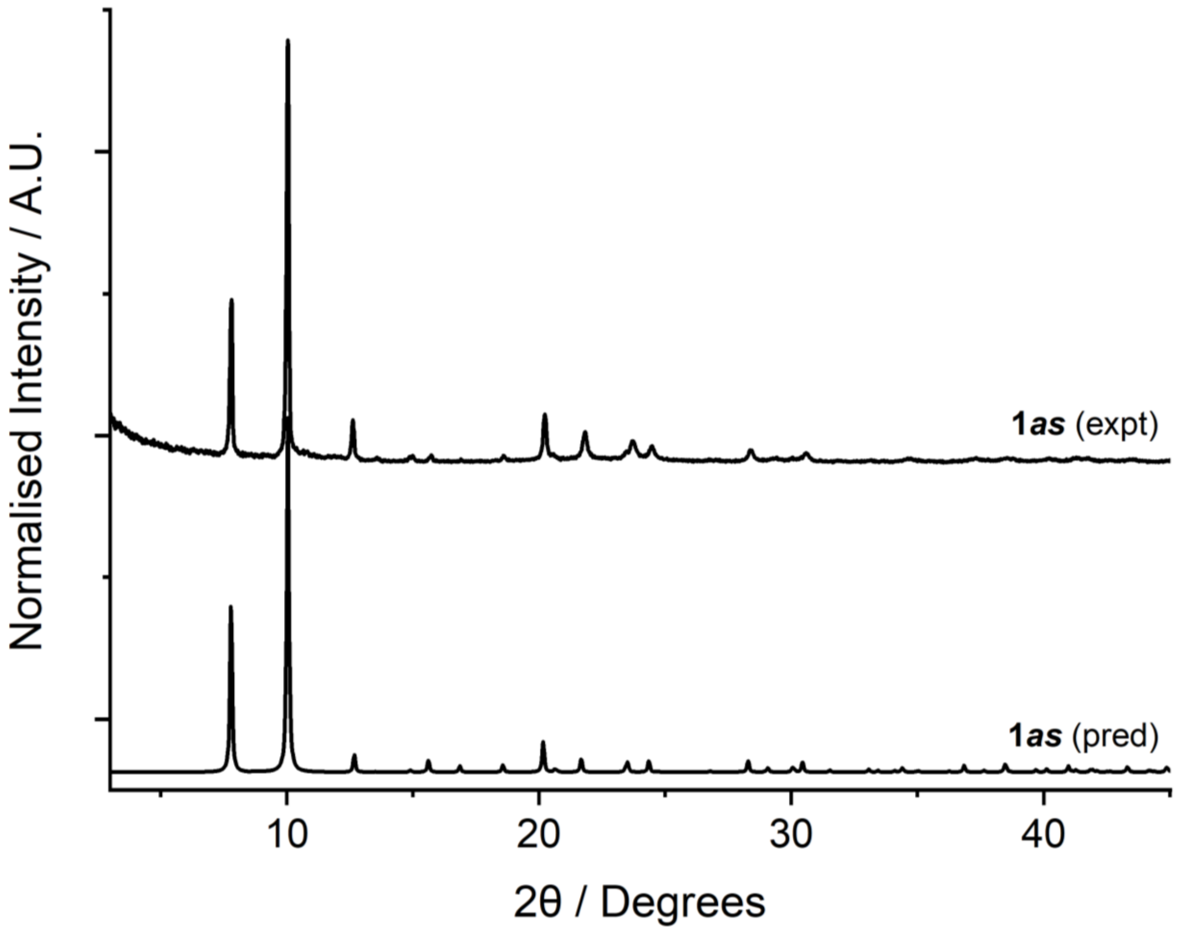

Figure S1. Stacked PXRD patterns of **1*as***, comparing the experimental diffractogram to that predicted for **1*as*** from its single crystal structure.

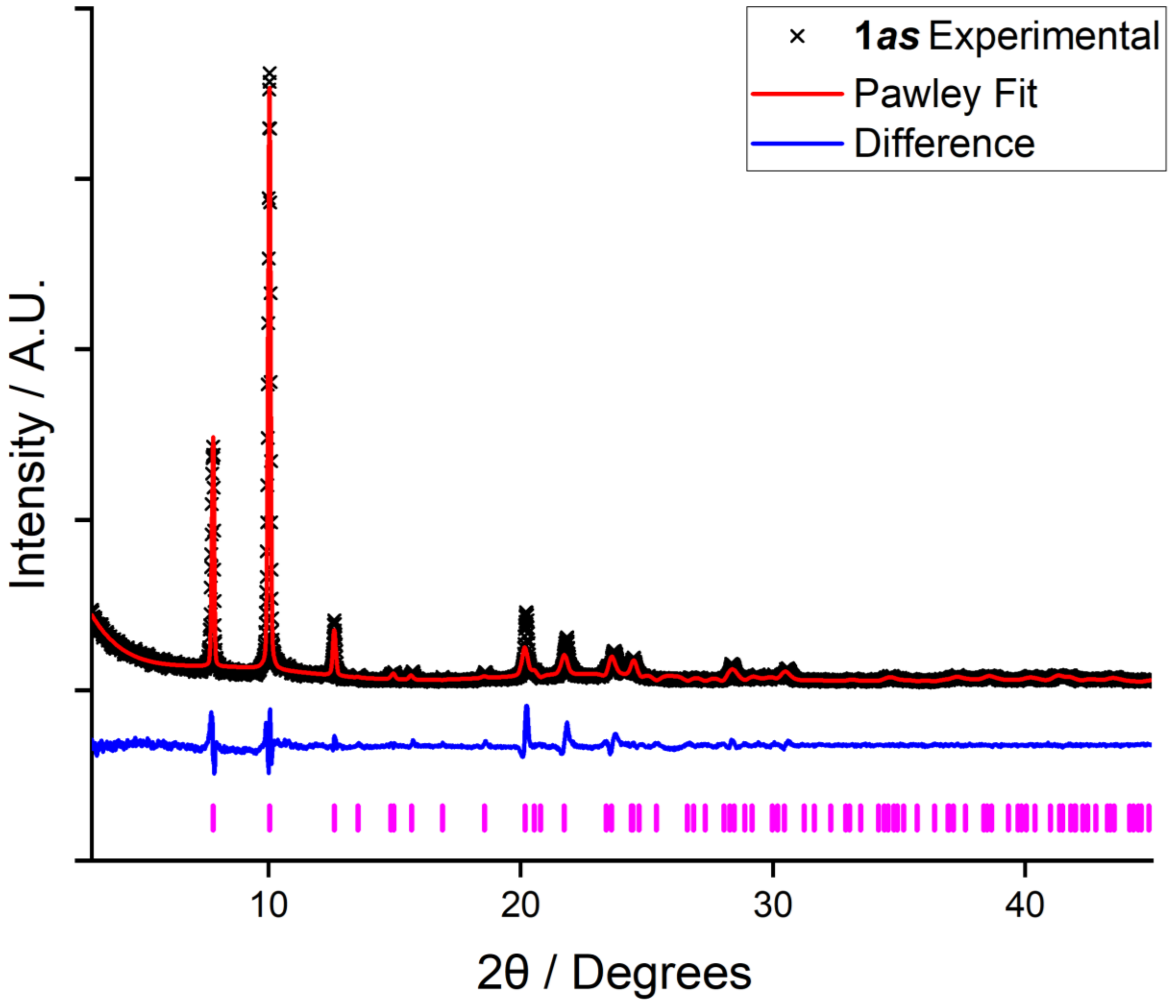

Figure S2. Pawley fit for the experimental PXRD pattern of **1*as***. Pawley fit yields hexagonal unit cell parameters; $a = b = 13.050(17)$, $c = 14.022(2)$ Å, $V = 2068(3)$ Å^3 ($R_{wp} = 13.86\%$).

SEM images show clearly the characteristic morphology associated with **1*as***. When dried from DMF, the samples retain their hexagonal rod shape and present as large 30 µm crystals, further indicating the phase-purity of the bulk sample (Figure S3).

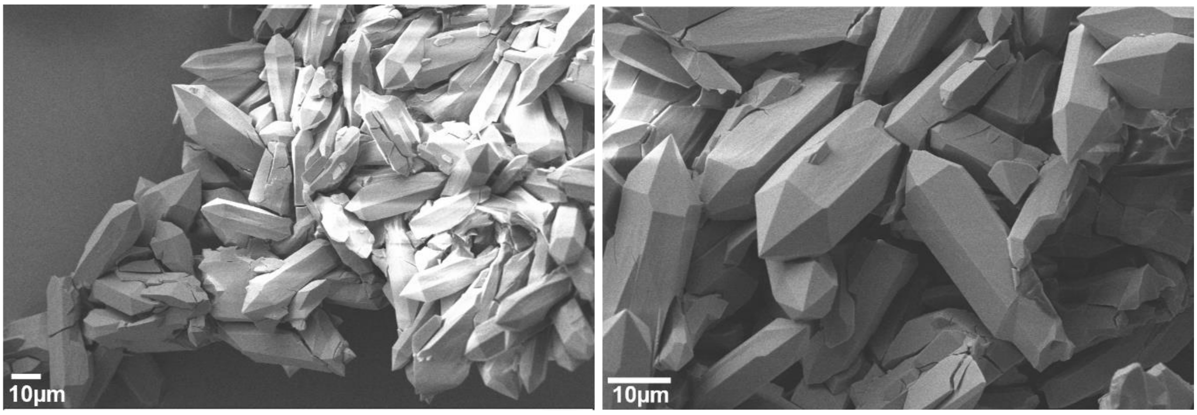

Figure S3. SEM images of **1*as*** dried from *N*,*N*-dimethylformamide at room temperature.

S3. Flexibility of 1*as* Under Drying

To examine the possibility of collecting crystal structures of **1** during the drying process, crystals of **1*as*** were mounted on MiTeGen loops, and crystallographic data were collected as the DMF solvent was allowed to diffuse out of the framework at room temperature. Unfortunately, mechanical degradation occurred as the solvent left the pores, meaning only two datasets could be collected, which we have termed **1*int-1*** and **1*int-2***.

Data quality for **1*int-1*** allowed refinement of a structure but with an R_1 of around 25% even after application of the SQUEEZE[S5] function in PLATON.[S6] This established that the connectivity of **1*as*** was maintained, but the data did not yield a publishable structure refinement. The unit cell parameters for **1*int-1*** did, however, confirm that the structure had begun to "close" as the DMF solvent left the pores, with an 11.2% decrease in unit cell volume compared to **1*as***.

Crystal data for 1*int-1*: Hexagonal; a = 12.030(2) Å, c = 14.687(2) Å, V = 1840.6(7) Å^3; T = 150 K; space group $P6_3/m$.

Data quality for **1*int-2*** were only sufficient to obtain unit cell parameters, showing an 18% decrease in unit cell volume compared to **1*as***.

Crystal data for 1*int-2*: Hexagonal, a = 11.532(6) Å, c = 14.764(11) Å, V = 1700(3) Å^3, T = 150 K; space group $P6_3/m$.

In both cases, comparison with previously reported "closed" structure for MIL-88A(Fe), where V = 1135 Å^3,[S7] confirms that neither dataset corresponds to a sample of **1** that is fully closed.

Given the known flexibility of MIL-88 materials, solvent loss from the framework can be visualised using PXRD. Notably, when **1*as*** is dried at room temperature in air from DMF, the change in structure can be monitored (Figure S4). Broad Bragg reflections result in difficulty indexing the patterns, however, a distinct shift to higher 2θ angles again suggests that **1*as*** is closing to a narrower pore framework as DMF diffuses from the pores.

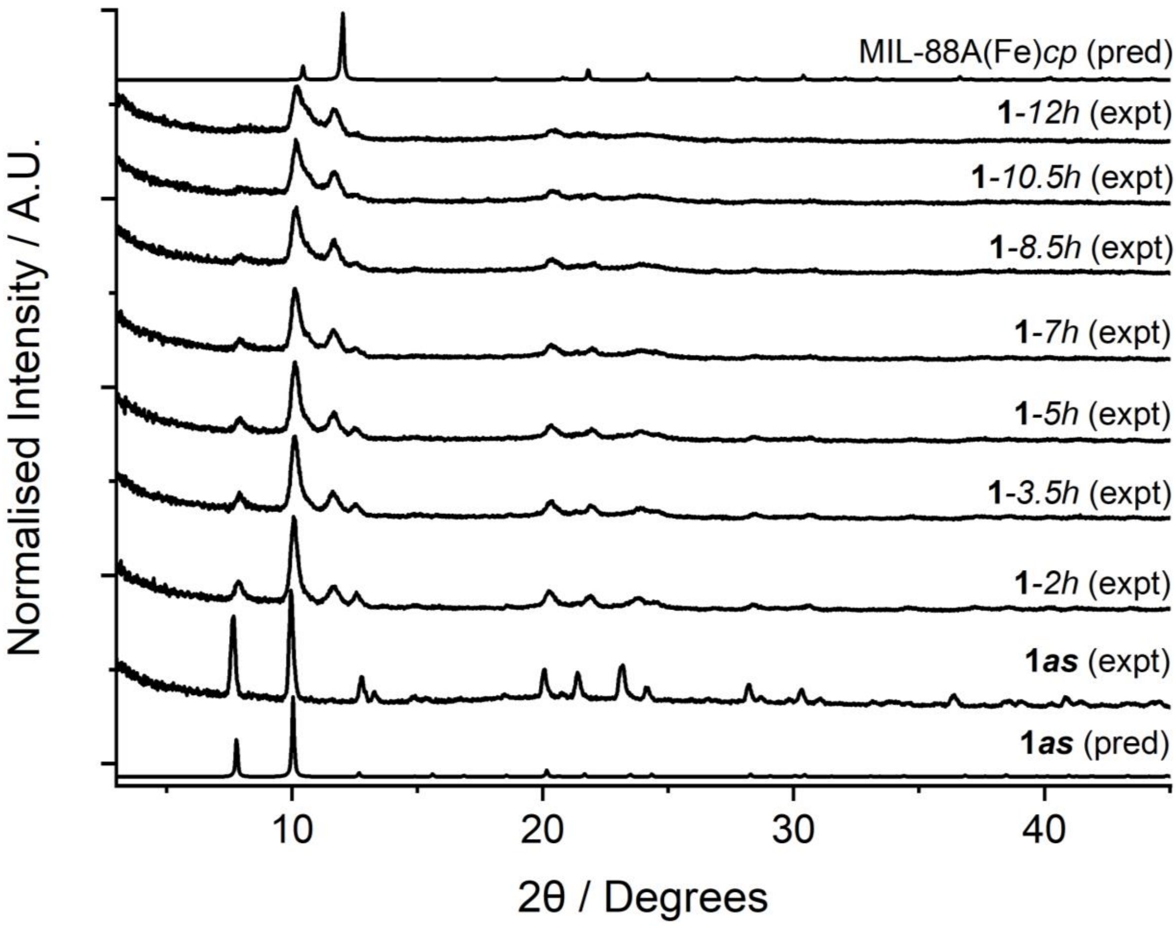

Figure S4. PXRD patterns of **1*as*** in DMF, obtained over 12 hours during *in situ* exposure to air (h = hours), compared to the simulated pattern for MIL-88A(Fe) closed pore.[S8]

S4. Solvent Exchange of 1*as*

To investigate the effect of solvent exchange on the framework, samples of **1*as*** were washed three times with fresh solvent and subsequently soaked in this solvent for 7 days. The bulk powder product was collected by centrifugation (4500 rpm, 10 minutes) and washed three times with fresh solvent. The samples, named **1*solvent***, were then dried at room temperature in a desiccator, followed by 4 hours in an isothermal oven (100 °C). Powder X-ray diffraction data for the samples are presented in Figure S5.

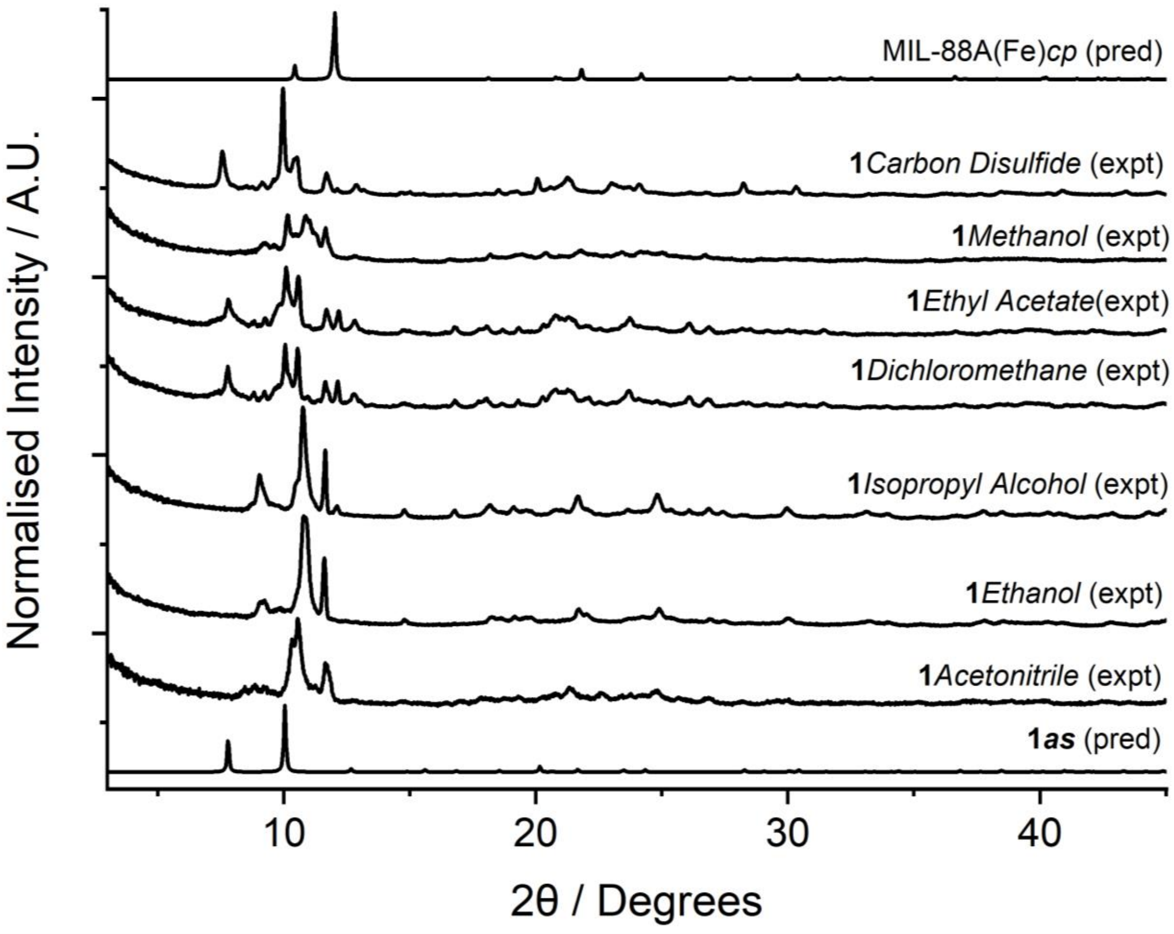

Figure S5. Stacked PXRD patterns of **1*solvent***, dried following solvent exchange for 1 week in various solvents.

A tentative Pawley fit was applied to index the experimental pattern of **1*Ethanol***, yielding hexagonal unit cell parameters $a = b = 11.096(46)$, $c = 15.264(8)$ Å, $V = 1628(8)$ Å^3 (R_{wp} = 11.98%, Figure S6). This provides an indication of the extent of contraction that the sample undergoes following solvent exchange (21.5% contraction in unit cell volume compared to **1*as***).

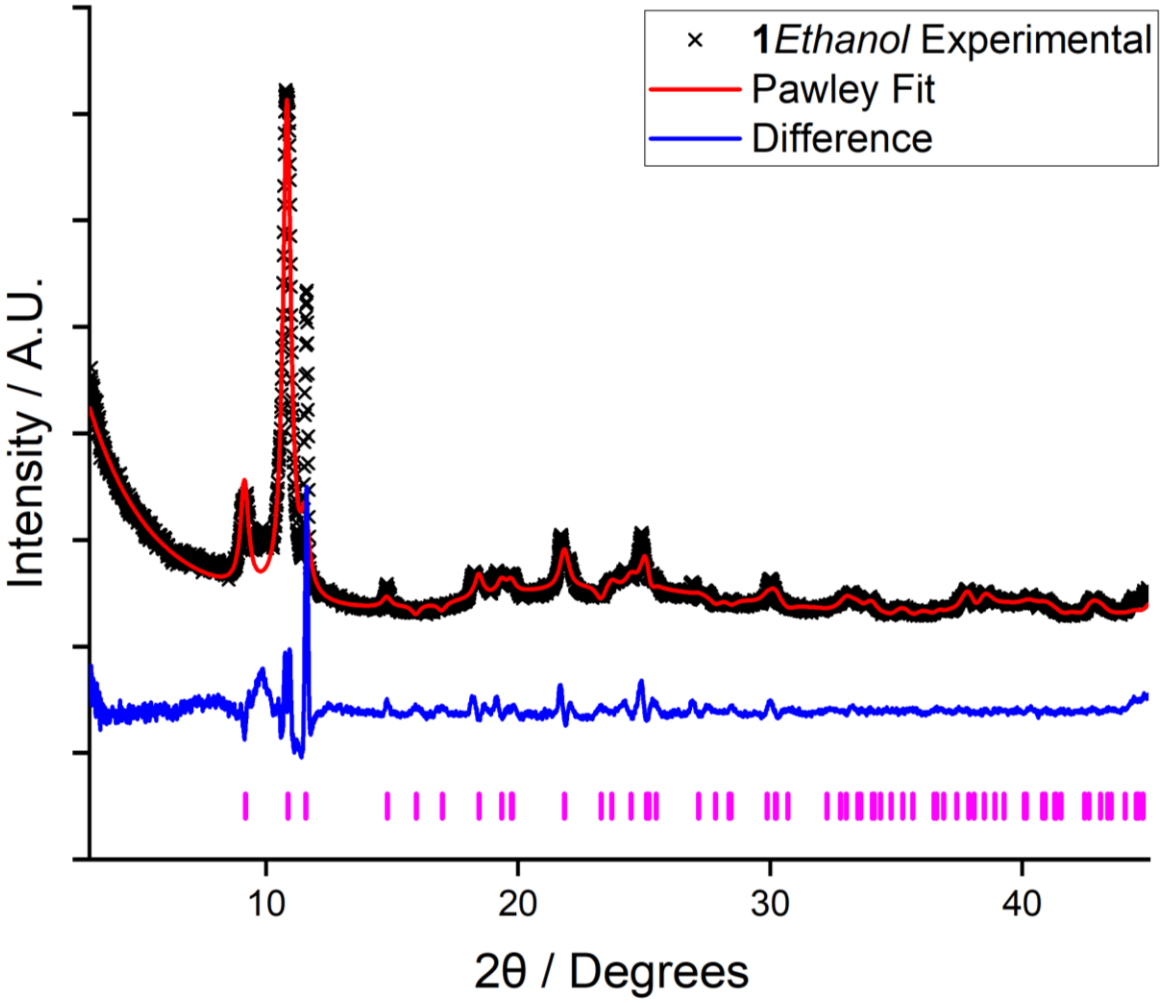

Figure S6. Tentative Pawley fit of the bulk experimental PXRD pattern of **1*Ethanol***. Pawley fit suggests hexagonal unit cell parameters; $a = b = 11.096(46)$, $c = 15.264(8)$ Å, $V = 1628(8)$ Å^3 (R_{wp} = 11.98%).

Regardless of solvent choice, ^{1}H NMR spectroscopic analysis of digested samples shows that DMF cannot be removed from the pores of **1*as*** with direct solvent exchange and drying (Figure S7).

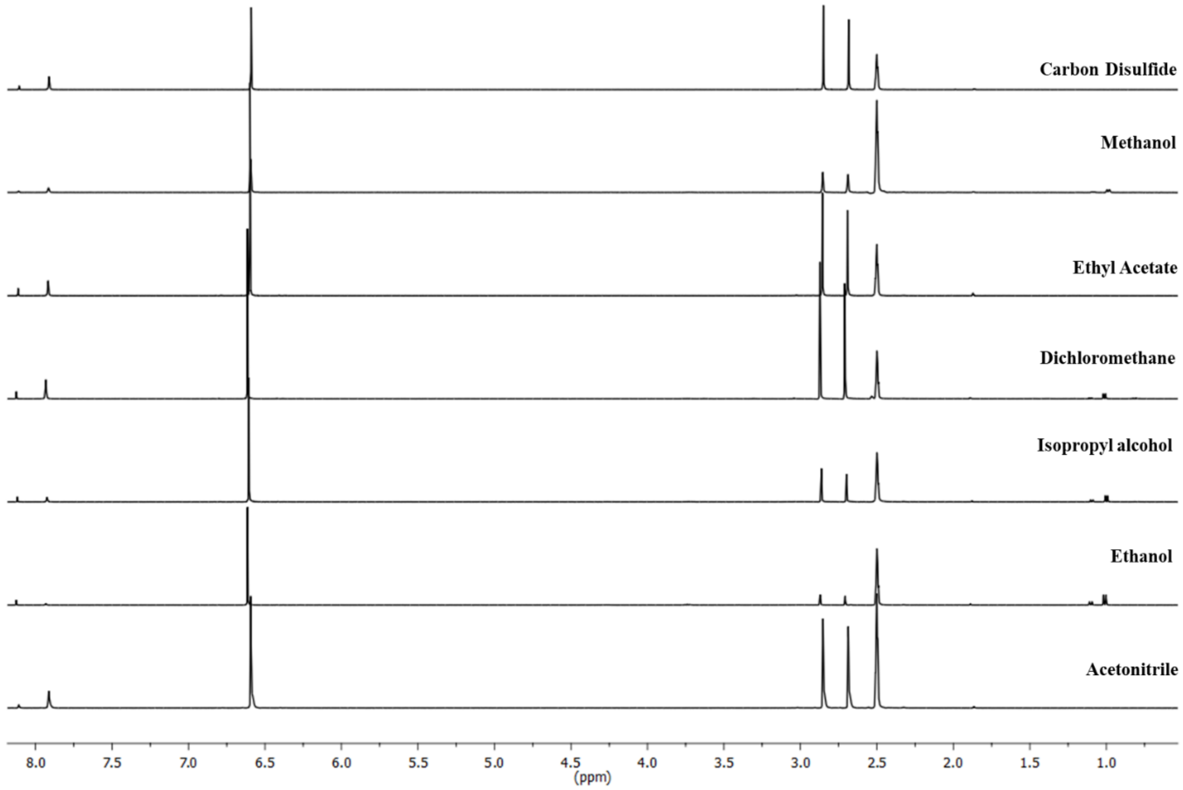

Figure S7. Stacked ^{1}H NMR spectra (DMSO-d_6 / D_2SO_4) of different digested samples of **1*solvent***, following solvent exchange for 1 week in various solvents. Resonances assigned to the methyl protons of DMF are clearly visible at δ ~2.7 and 2.85 ppm.

Density functional theory (DFT) calculations were used to generate a range of simulated structures of **1** with different unit cell volumes and thus with different extents of "closing". All DFT calculations were performed using the CP2K code (version 8.2), which uses a mixed Gaussian/plane-wave basis set.[S9] We employed double-z polarization quality Gaussian basis sets[S10] and a 400 Ry plane-wave cutoff for the auxiliary grid, in conjunction with the Goedecker-Teter-Hutter pseudopotentials.[S11] All DFT calculations were performed in the G-point approximation with sufficiently large supercells. Total energy calculations and structural optimizations, including both atomic coordinates and cell parameters, were performed under periodic boundary conditions at the DFT level using the PBE exchange and correlation functional,[S12] with Grimme's D3 van der Waals correction (PBE+D3).[S13] A convergence threshold of 1.0×10^{-6} Hartree was used for the self-consistent field cycle, and structural optimizations were considered to have converged when the maximum force on all atoms falls below 4.5×10^{-4} Hartree/Bohr.

Starting from the experimentally resolved open-pore crystal structure **1*as***, we first computationally optimised the structure at DFT level. We then further optimised the open-pore structure of **1** under pressure, which forced the structure to undergo the open to closed phase transformation, after which we removed the pressure and re-optimised the closed-pore structure to obtain the lattice parameters of the closed-pore structure, **1*cp-sim***, under ambient pressure. We note our dispersion corrected DFT may overestimate the density of **1*cp-sim***.

To better interpret the experimental diffraction data, we generated a range of simulated structures of **1** with different cell volumes. Starting from **1*cp-sim***, we selected a series of crystallographic *a/b* axis parameters between 9.485 Å and 13.485 Å, which correspond approximately to the *a/b* axes of the open-pore and closed-pore structures, respectively. We then performed partial cell optimisations, i.e., only allowing the crystallographic *c* axis parameter to relax while keeping the *a/b* parameters fixed during the cell optimisation and, from this, we generated a series of simulated structures of **1** with unit cell volumes ranging from 1213 $Å^3$ to 2126 $Å^3$. The naming scheme for these simulations is **1*sim*** (*a* = x), where x = the fixed *a* axis parameter. From these structures, powder X-ray diffractograms were predicted (Figure S8) and used to qualitatively assess the experimental diffraction data. Unit cell parameters for all simulated structures are given in Table S1, and plots of the *a* axis parameter versus unit cell volume and the *c* axis parameter are given in Figure S9 and Figure S10 to show the continuity of the breathing of the structure during the simulations.

Selected experimental diffractograms of samples of **1** are compared to pertinent predicted diffractograms from simulated structures in Figure S11 to qualitatively assess the level of opening of the MOF.

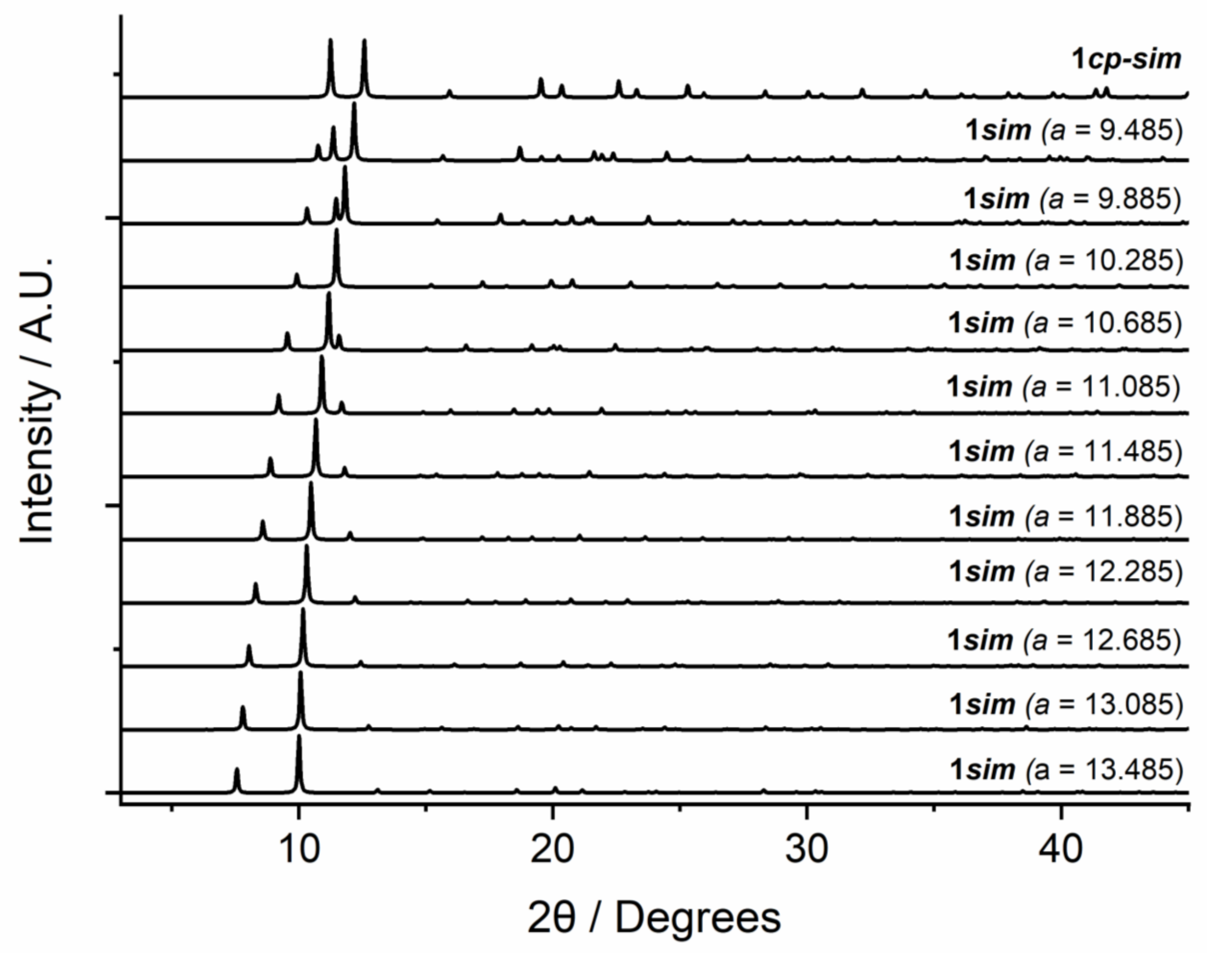

Figure S8. Stacked predicted PXRD patterns of **1*sim*** (*a* = x) derived from DFT simulations. Unit cell data for the simulations are provided in Table S1.

Table S1. Unit cell parameters for DFT simulated structures of 1.

**MOF**	***a* axis / Å**	***c* axis / Å**	***V* / Å^3**
**1*cp-sim***	9.0849	15.7156	1123.32
**1*sim* (*a* = 9.485)**	9.4850	15.5761	1213.57
**1*sim* (*a* = 9.885)**	9.8850	15.4228	1305.11
**1*sim* (*a* = 10.285)**	10.2850	15.3762	1408.60
**1*sim* (*a* = 10.685)**	10.6850	15.2644	1509.24
**1*sim* (*a* = 11.085)**	11.0850	15.1326	1610.33
**1*sim* (*a* = 11.485)**	11.4850	14.9814	1711.37
**1*sim* (*a* = 11.885)**	11.8850	14.7188	1800.53
**1*sim* (*a* = 12.285)**	12.2850	14.4793	1892.47
**1*sim* (*a* = 12.685)**	12.6850	14.2264	1982.47
**1*sim* (*a* = 13.085)**	13.0850	13.8756	2057.45
**1*sim* (*a* = 13.485)**	13.4850	13.5040	2126.64

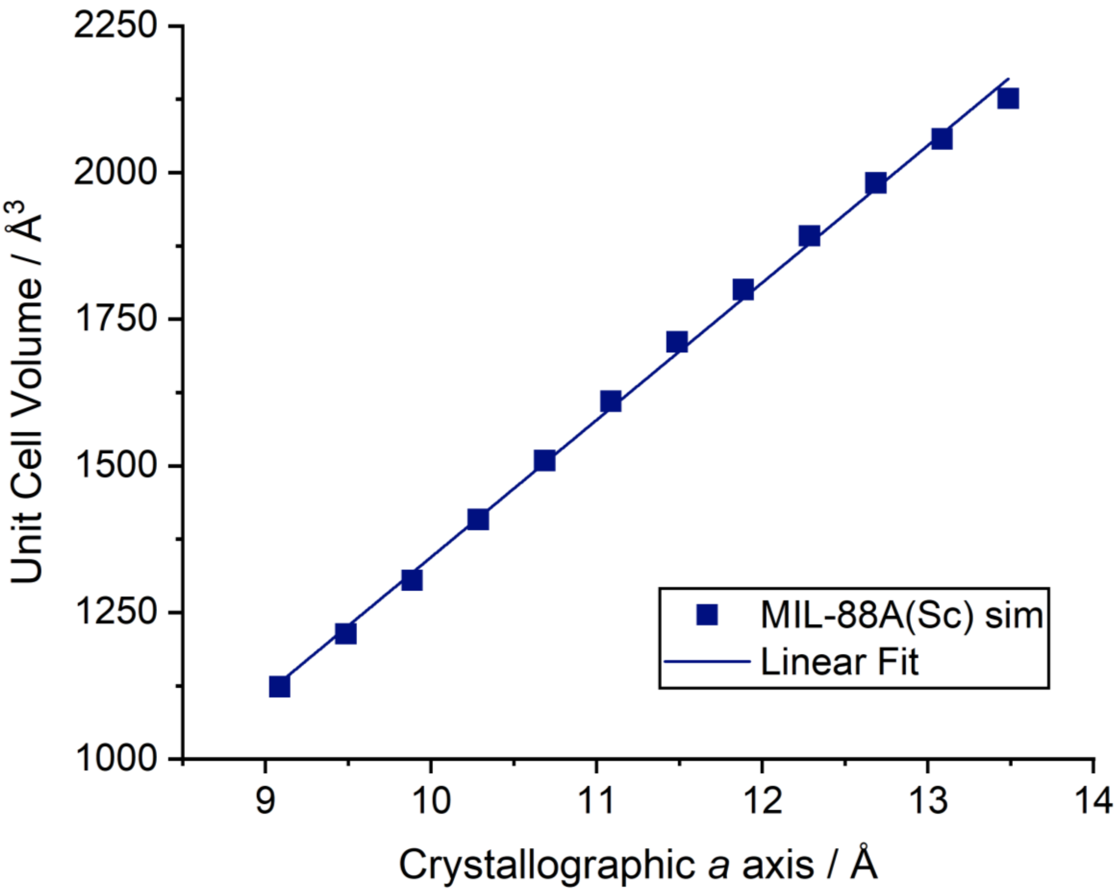

Figure S9. Plot of unit cell volume vs crystallographic *a* axis for the DFT-optimised **1*sim*** structural models.

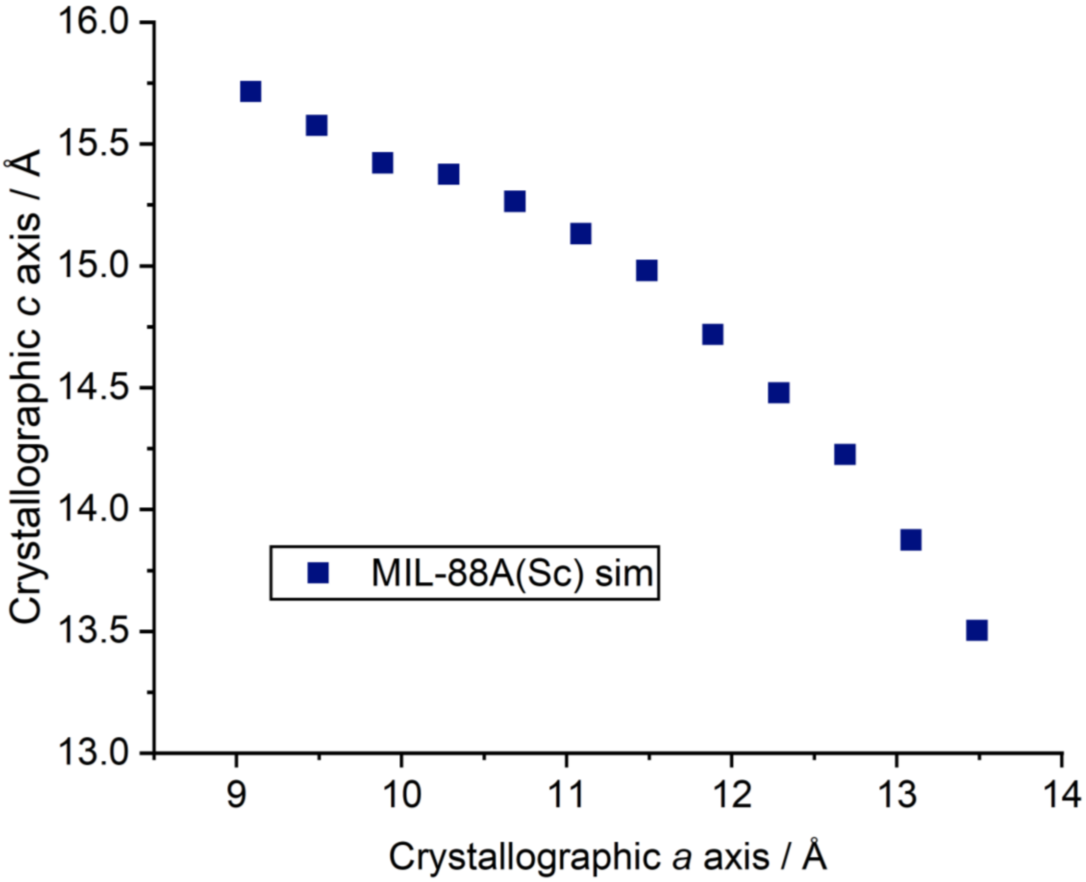

Figure S10. Plot of crystallographic *c* axis vs crystallographic *a* axis for the DFT-optimised **1*sim*** structural models.

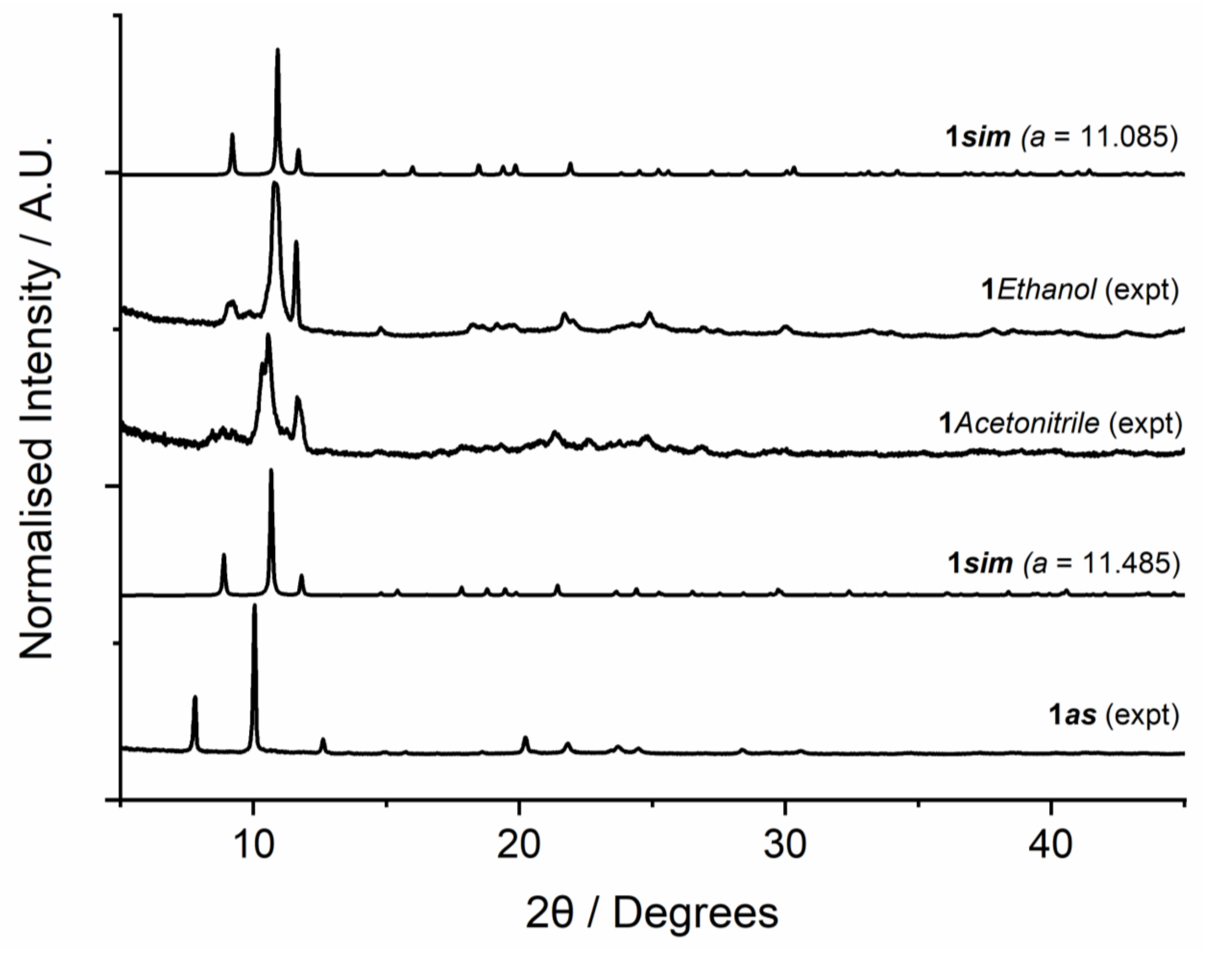

Figure S11. Stacked PXRD patterns of **1*as*** and **1***solvent* compared to the predicted patterns for simulated structures of **1*sim*** where the *a* axis has been set at 11.485 Å and 11.085 Å, respectively, and the structure optimised by DFT.

The exception to these solvent exchange trends is water; a sample of **1*as*** soaked in water for 1 hr resulted in a structural opening (Figure S12), with a Pawley fit giving unit cell parameters of $a = b = 14.589(82)$ Å, $c = 13.077(3)$ Å, $V = 2410(16)$ Å^3 ($R_{wp} = 13.79\%$, Figure S13). This corresponds to a 16.3% *increase* in unit cell volume compared to **1*as***. However, prolonged solvation of the sample in water led to structural breakdown.

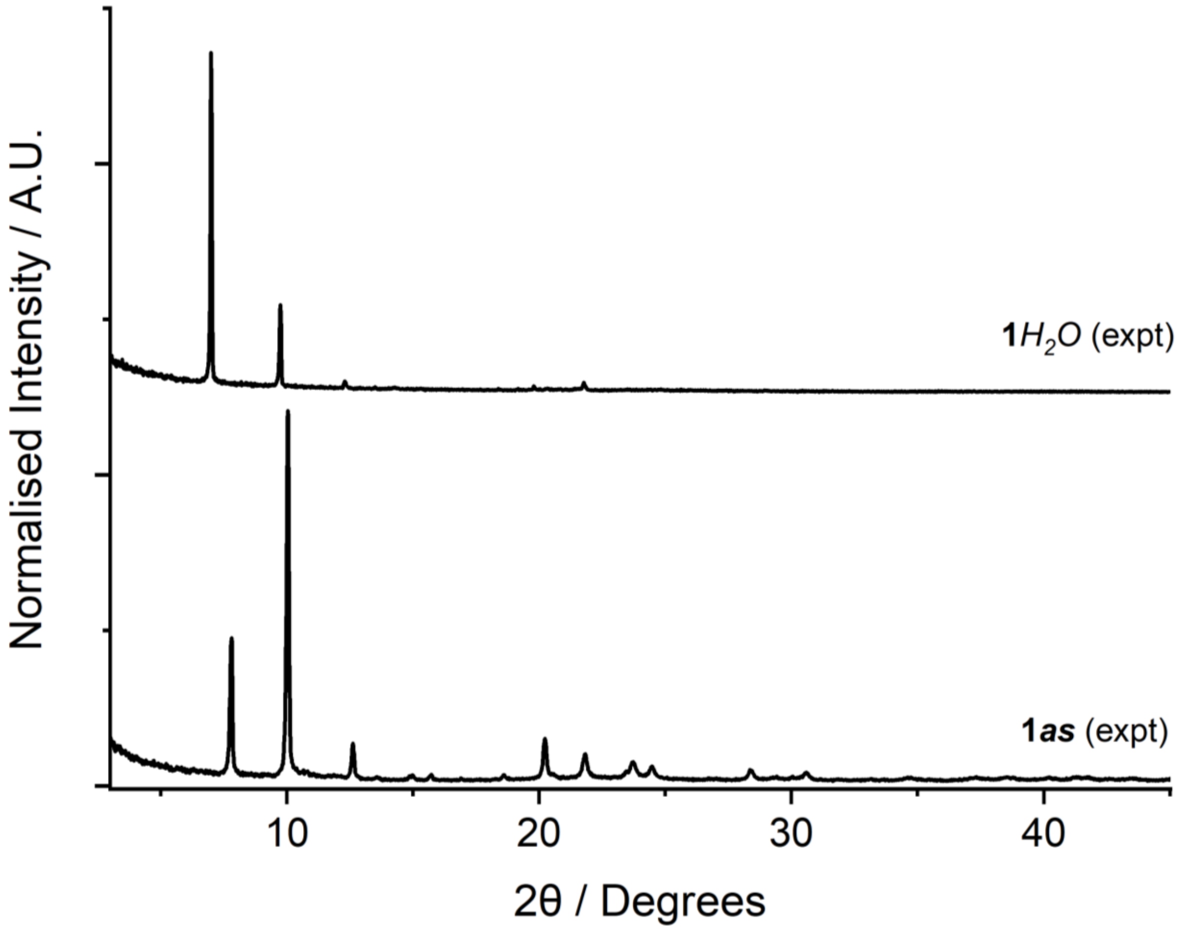

Figure S12. Stacked PXRD patterns of **1*as*** compared with **1**H_2O; where **1**H_2O represents a sample of **1*as*** following a 1 hr soak in deionised water.

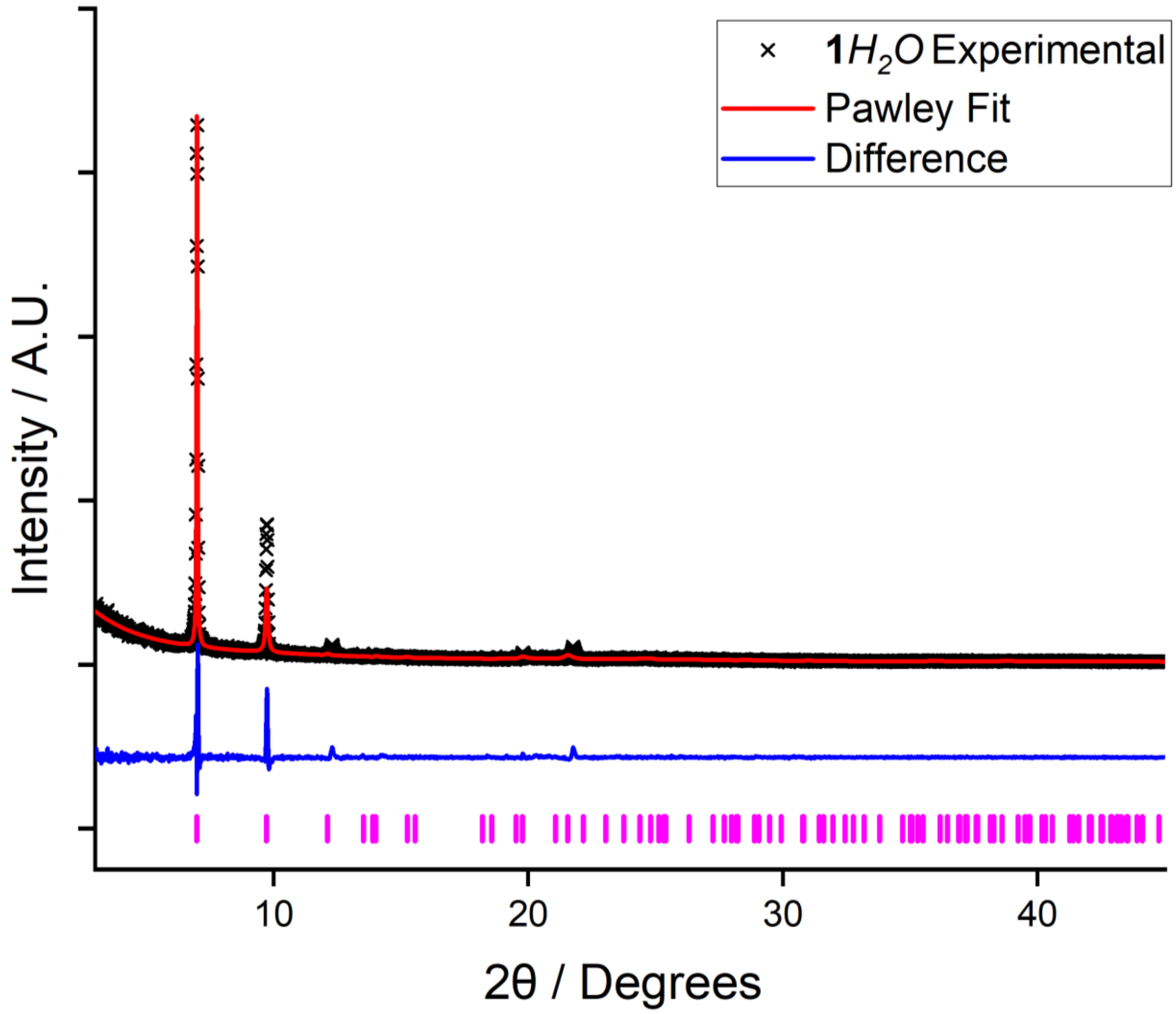

Figure S13. Pawley fit of experimental **1***H₂O* PXRD pattern. Pawley fit yields hexagonal unit cell parameters; $a = b = 14.589(82)$ Å, $c = 13.077(3)$ Å, $V = 2410(16)$ Å^3 ($R_{wp} = 13.79\%$).

To compare the flexibility of **1** with the existing MIL-88A(Fe) material, unit cell parameters have been plotted against each other for all available experimental data sets and **1*cp-sim***. A plot of the unit cell volume (V) versus the crystallographic a axis is given in the manuscript (Figure 3), while plots of V versus c axis and c axis versus a axis are given in Figure S14 and Figure S15. Data are tabulated in Table S2.

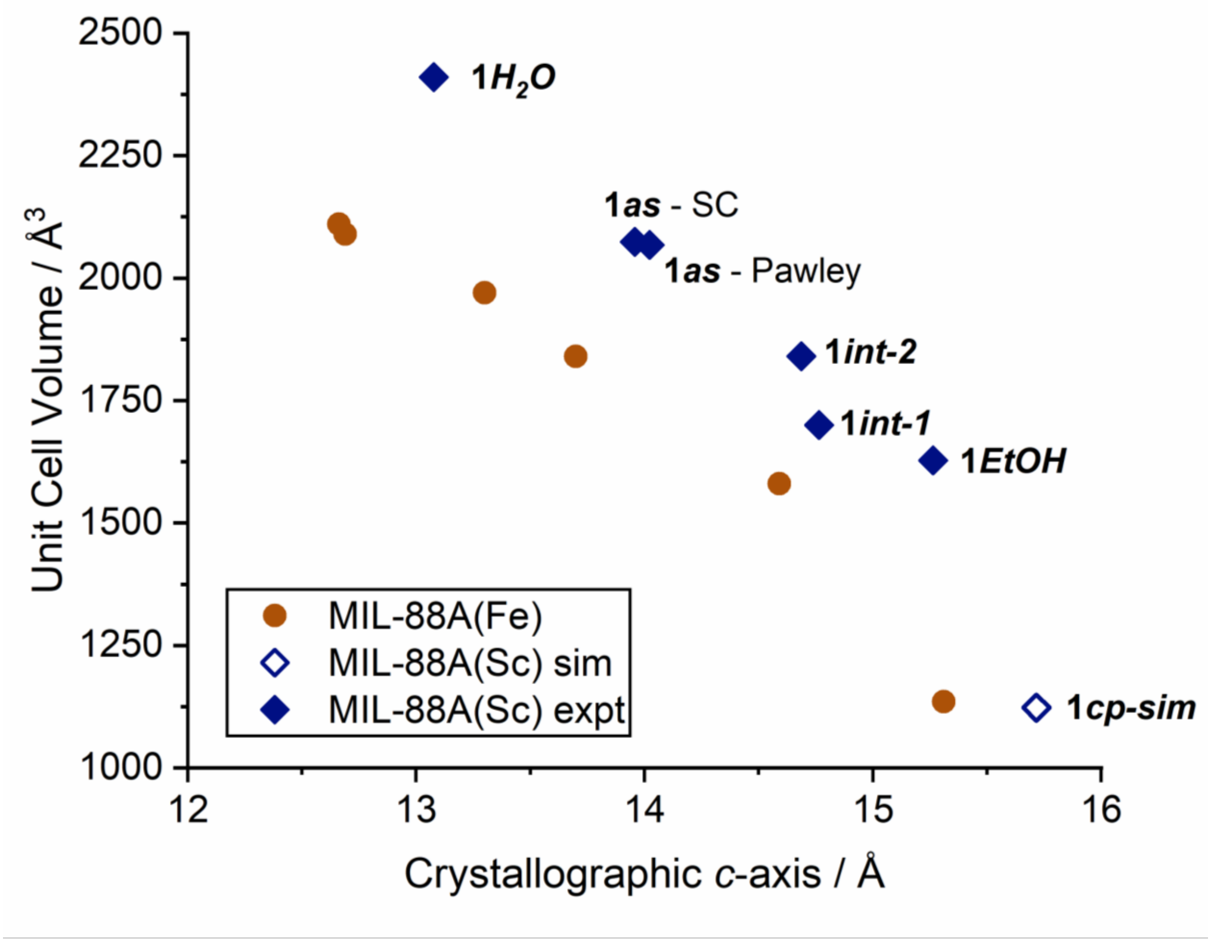

Figure S14. Plot of unit cell volume vs crystallographic *c* axis for varying datasets collected for MIL-88A(Sc) (**1**) compared to data previously published for MIL-88A(Fe).[S7]

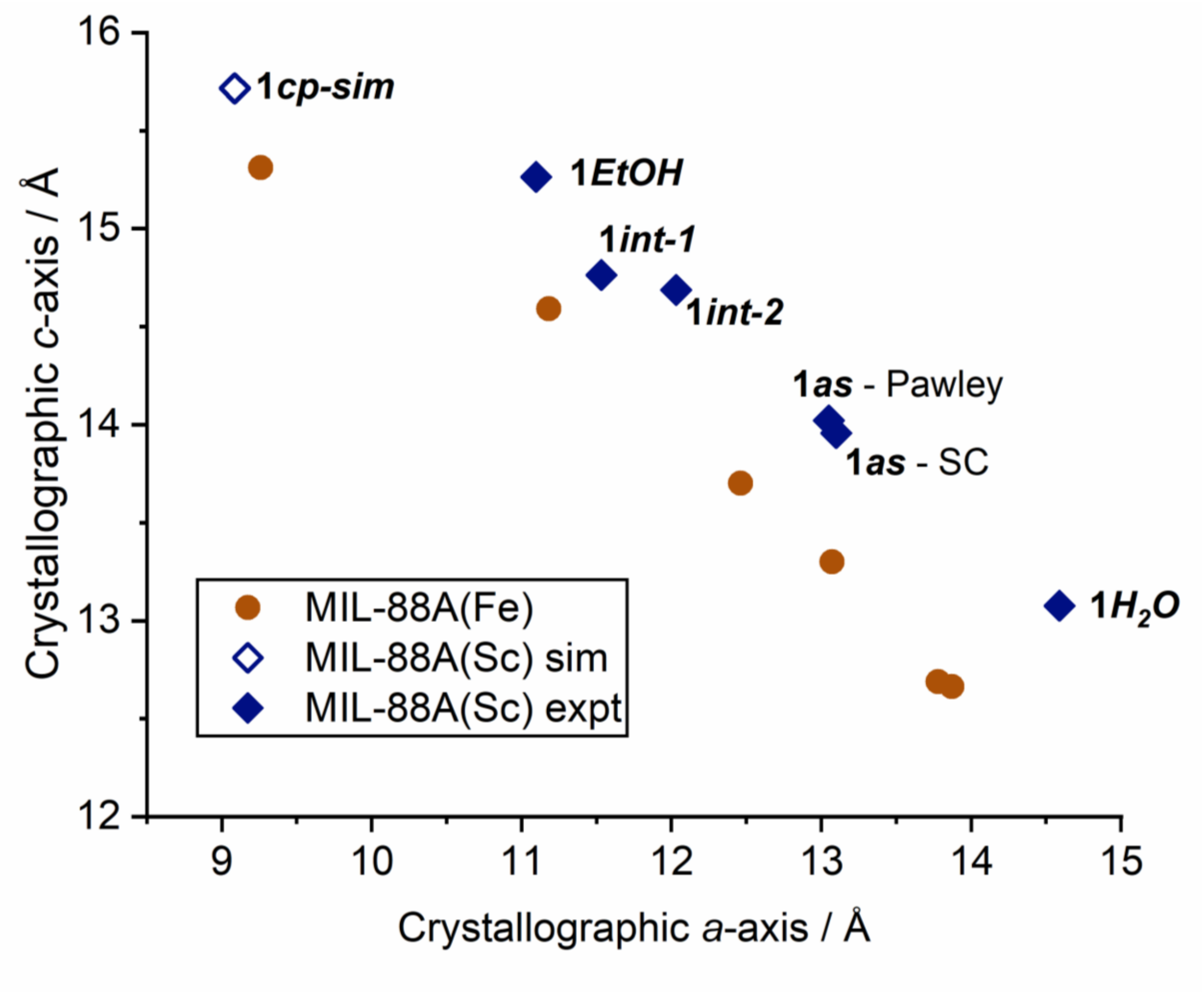

Figure S15. Plot of crystallographic *c* axis vs crystallographic *a* axis for varying datasets collected for MIL-88A(Sc) (**1**) compared to data previously published for MIL-88A(Fe).[S7]

Table S2. Collated data used for Figures 3, S14, and S15. Data for MIL-88A(Fe) taken from previously published work.[S7]

**MOF**	**Data Type**	***a* axis / Å**	***c* axis / Å**	***V* / Å^3**
**1*H$_2$O***	Pawley fit	14.589(82)	13.077(3)	2410(16)
**1*as* - SC**	Single crystal	13.098(3)	13.958(4)	2073.8(11)
**1*as* - Pawley**	Pawley fit	13.050(17)	14.022(2)	2068(3)
**1*int-1***	Single crystal	12.030(2)	14.687(2)	1840.6(7)
**1*int-2***	Single crystal	11.532(6)	14.764(11)	1700(3)
**1*EtOH***	Pawley fit	11.096(46)	15.264(8)	1628(8)
**1*cp-sim***	DFT	9.0849	15.7156	1123.32
MIL-88A(Fe) H_2O	Rietveld refinement	13.871(1)	12.663(1)	2110.0(2)
MIL-88A(Fe) MeOH	Simulation	13.78	12.69	2090
MIL-88A(Fe) EtOH	Simulation	13.07	13.30	1970
MIL-88A(Fe) BuOH	Simulation	12.46	13.70	1840
MIL-88A(Fe) as-synthesised	Simulation	11.18	14.59	1580
MIL-88A(Fe) dried	Simulation	9.26	15.31	1135

S5. Characterisation of 1*np*

For reasons of solvent compatibility with postsynthetic modification conditions, the MeCN washed material was taken forward for bromination and named **1*np***.

1*np* was collected by washing **1*as*** three times in acetonitrile (MeCN) and drying in a desiccator, under vacuum followed by 4 hours in an isothermal oven (100 °C). Elemental analysis, specifically N content, suggested 1.5 moles of DMF solvent persisted per Sc_3O cluster. Elemental analysis calculated for $[Sc_3O(C_4H_2O_4)_3(H_2O)_2(OH)]\cdot 1.5DMF$: C, 30.22%; H, 3.31%; N, 3.20%. Found: C, 31.89%; H, 3.43%; N, 3.23%.

Similarly, ^{1}H NMR spectroscopic analysis of an acid-digested bulk sample of **1*np*** suggested 1.5 molecules of DMF per Sc_3O cluster (Figure S16), and no retention of MeCN.

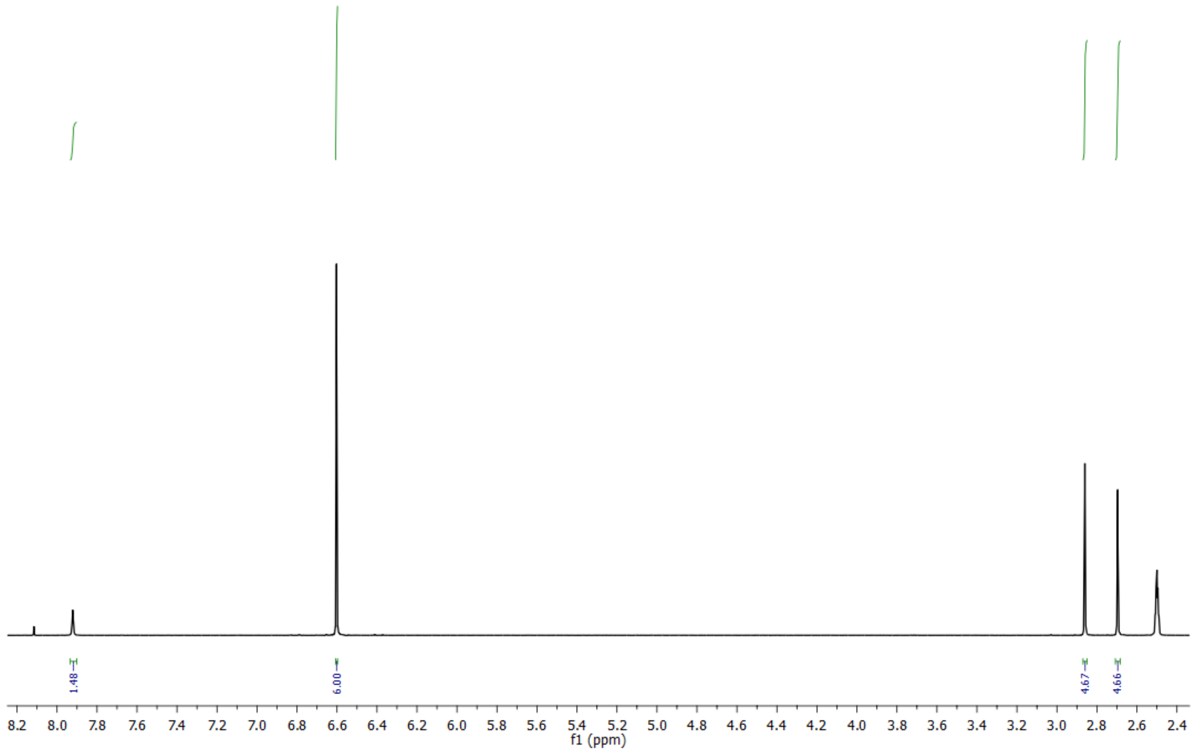

Figure S16. Partial ^{1}H NMR spectrum (DMSO-d_6 / D_2SO_4) of **1*np***. δ/ppm: 2.69 (s, 5.3H), 2.85 (s, 5.5H), 6.59 (s, 6H), 7.91 (s, 1.5H). Taking an average integral of 4.67 for the DMF methyl groups gives a fumarate to DMF ratio of 3:1.56, which corresponds well with the 1.5 moles DMF confirmed by elemental analysis.

Thermogravimetric analysis (TGA) has been used to assess the thermal stability of **1*np***, which shows reasonable thermal stability until approximately 360 °C when the framework begins to break down (Figure S17). The resultant residual mass is 29.4% wt, and when the TGA is scaled to remove any weakly adsorbing residues below 100°C, the resultant residual mass is 31.0% wt. This is accordant with the expected residual mass of Sc_2O_3 (31.5% wt) calculated from the formula $[Sc_3O(C_4H_2O_4)_3(H_2O)_2(OH)(C_3H_7NO)_{1.5}]$ and corresponds well with the elemental analysis results, where N analysis suggests the presence of 1.5 DMF molecules per scandium cluster.

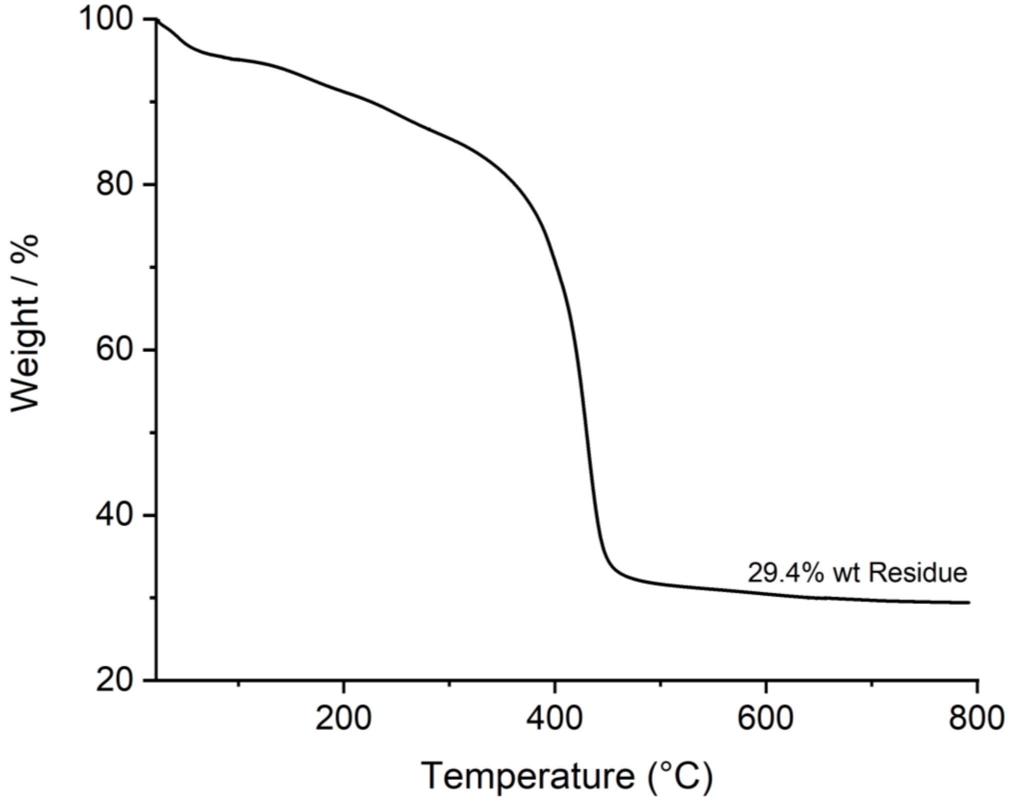

Figure S17. TGA profile of **1*np***, yielding a 29.4% wt residue of Sc_2O_3. When scaled to remove the weakly adsorbed guests (e.g. atmospheric water) that are lost below 100 °C, the residual mass is 31.0% wt.

As a result of the exchange conditions used to yield **1*np***, the morphology of the sample has been altered compared to **1*as*** as seen by SEM imaging. Many rods appear broken and clumped together, due to mechanical fracture as the crystals contract on loss of solvent (Figure S18).

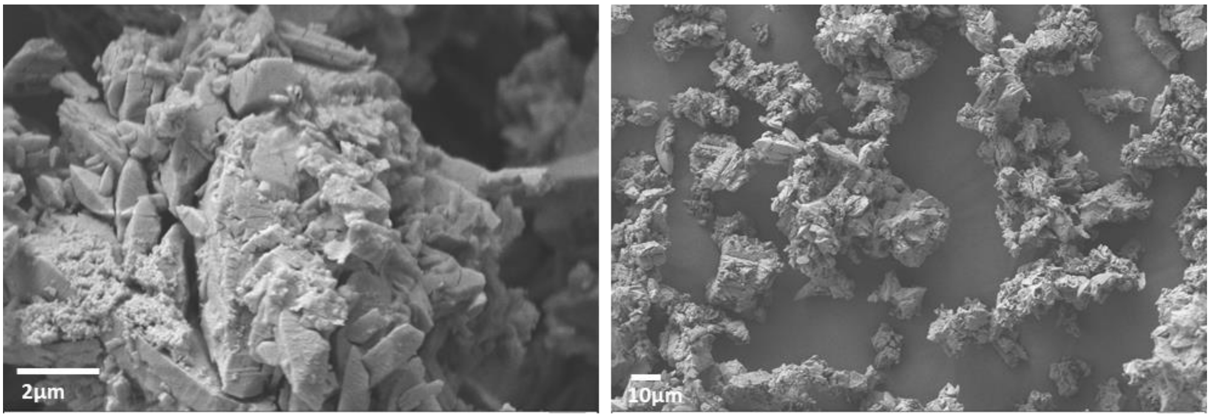

Figure S18. SEM images of **1*np***, dried from acetonitrile.

In order to determine the porosity of **1*np***, gas sorption isotherms were collected at a range of temperatures. Nitrogen gas adsorption and desorption isotherms at 77 K revealed that the framework is not porous to nitrogen gas. This is likely a combination of residual DMF blocking the pores and the framework not reopening under these conditions. However, uptake of CO_2 at 273 K, 288 K and 298 K (Figure S19) indicates that **1*np*** has a good affinity for CO_2 molecules between 0-1 bar and is likely opening to accommodate the gas within the pores. A moderate hydrogen adsorption of 1.3 wt% at 1 bar is also achieved for **1*np***.

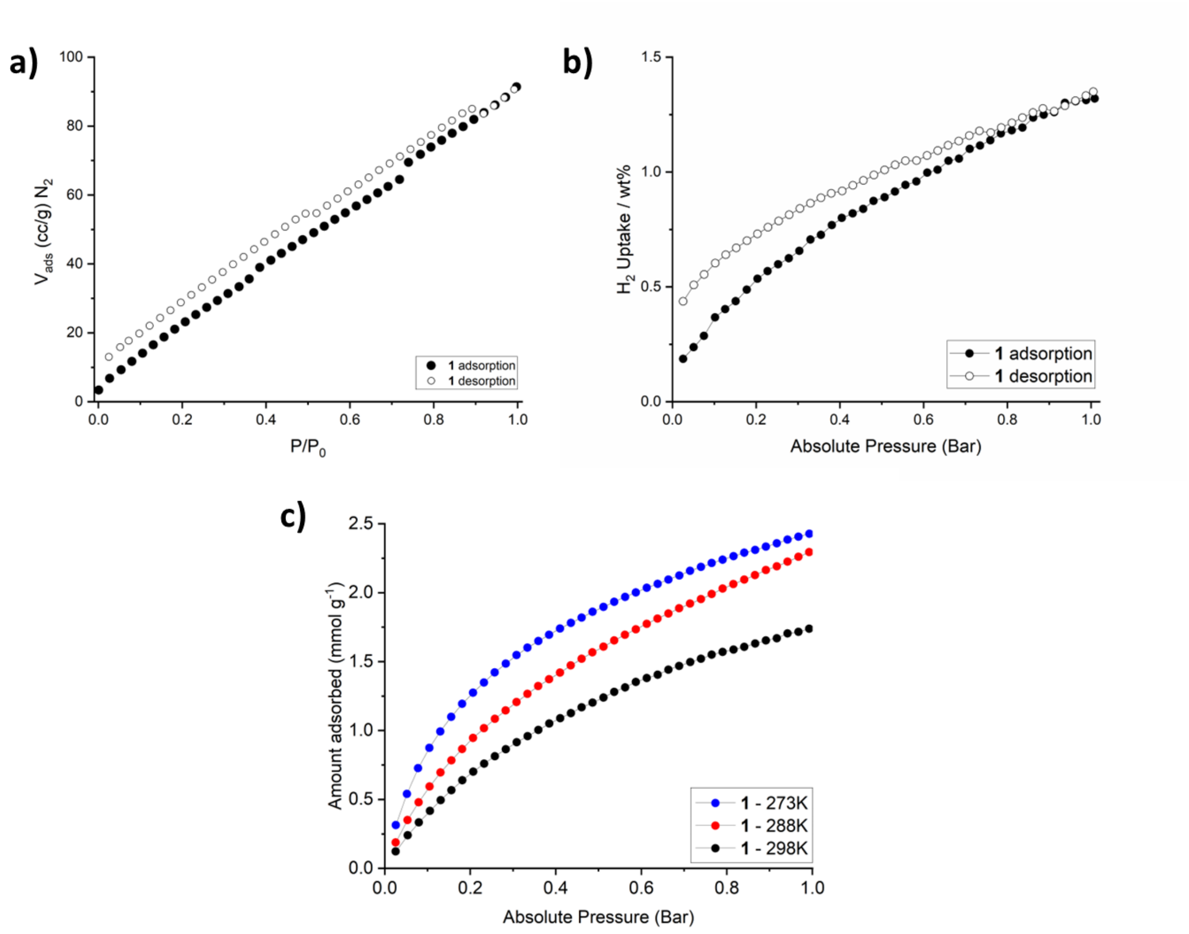

Figure S19. a) Nitrogen adsorption desorption isotherm for **1*np***, collected at 77 K No micropore adsorption is evident. b) H_2 adsorption isotherm for **1*np***, collected at 77 K. c) CO_2 adsorption isotherm for **1*np***, collected at 298 K (black), 283 K (red) and 273 K (blue).

S6. Synthesis and Characterisation of 2

Synthesis of [$Sc_3O(C_4H_2O_4)_3(HCO_2)(H_2O)_{0.5}(C_3H_7NO)_{0.5}$]·$(C_3H_7NO)_{0.5}$ (2): Fumaric acid (0.04 g, 0.35 mmol) and scandium nitrate hydrate (0.10 g, 0.33 mmol) were added to a 25 mL Pyrex vial and DMF (5 mL) was added. To this mixture, concentrated HCl (10 μL, 0.33 mmol) was added and the reaction mixture was sonicated for 10 minutes. The vial was placed in an isothermal oven at 150 °C for 24 hours. The vial was removed from the oven and allowed to cool to room temperature. Single crystals were collected by pipette from the mother solution and left to stand until further analysis. The bulk solid product was collected by centrifugation (4500 rpm, 10 minutes) and washed three times with fresh DMF. Prior to further analysis, **2** was washed with acetonitrile three times and dried in a desiccator at room temperature, followed by 4 hours in an isothermal oven (100 °C).

Crystal data for 2: $Sc_3O(C_4H_2O_4)_3(H_2O)_{0.5}(HCO_2)(C_3H_7NO)$, M_r = 620.17, crystal dimensions 0.01 x 0.04 x 0.08 mm, Orthorhombic; a = 17.0967(9) Å, b = 15.1239(9) Å, c = 12.2795(7) Å, V = 3175.1(3) Å^3, T = 150 K, space group Pnma (No. 62), Z = 4, 22749 reflections measured, 3134 independent reflections (R_{int} = 0.094) which were all used in calculations. The final R_1 = 0.055 for 2246 observed data $R[F^2 > 2\sigma(F^2)]$ and $wR(F^2)$ = 0.163 (all data). The SQUEEZE[S5] function in PLATON[S6] was used to account for pore solvent electron density, the solvent accessible volume was calculated as 599 Å^3 and containing 134 electrons per unit cell. CCDC deposition number 2150612.

2 has a similar SBU to **1**, however, where **1** is capped by two water molecules and a hydroxide, **2** has differing connections. Across three scandium centres, the axial linkers are two bridging formate (HCO_2^-) moieties and the final scandium unit alternates between water and DMF coordinated in 50% occupancies. The coordinated DMF ligand is itself disordered across two potential orientations with equal occupancy, related by symmetry (Figure S20a). Each water ligand has a further molecule of DMF hydrogen bonding to it (O10···O11 = 2.460(16) Å), which is also disordered over two positions with equal occupancy, related by symmetry (Figure S20b). A number of atoms from different disorder patterns are coincident, giving a complex overall model of disordered DMF molecules that are both coordinated to the cluster and resident in the pores. Nevertheless, this could be satisfactorily modelled to give the material the overall formula of [$Sc_3O(C_4H_2O_4)_3(HCO_2)(H_2O)_{0.5}(C_3H_7NO)_{0.5}$]·$(C_3H_7NO)_{0.5}$.

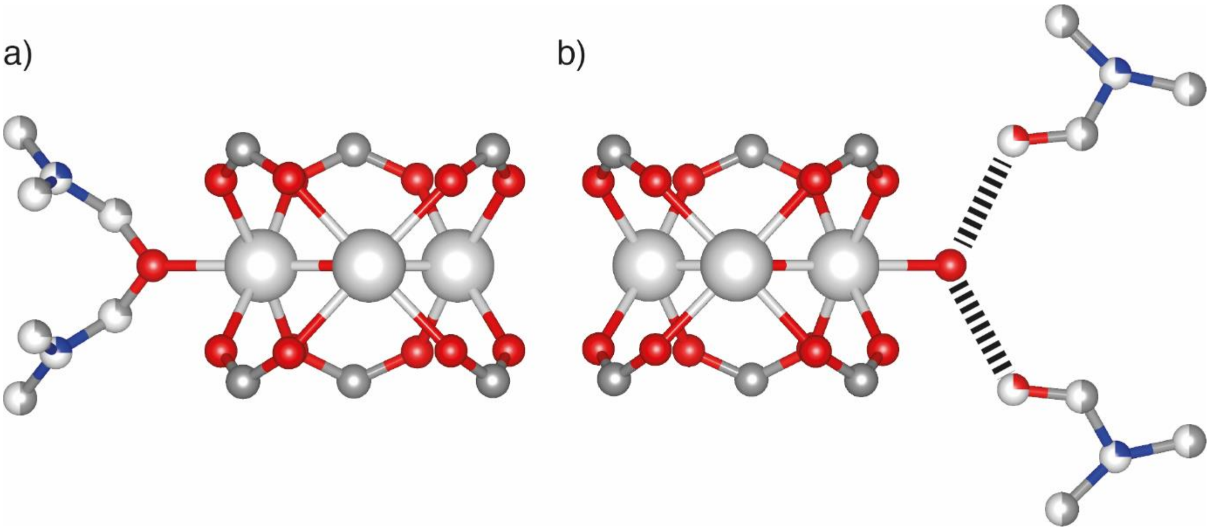

Figure S20. Disorder in **2**. a) The two orientations of cluster-bound DMF. b) The two-orientations of DMF hydrogen bonded (black line) to cluster-bound water.

Bulk phase purity was confirmed by a Pawley fit of experimental powder X-ray diffraction data (Figure S21) that gave unit cell parameters close to those obtained by single crystal X-ray diffraction. Samples dried from both DMF and MeCN were examined by SEM, showing different morphology to **1** (Figure S22).

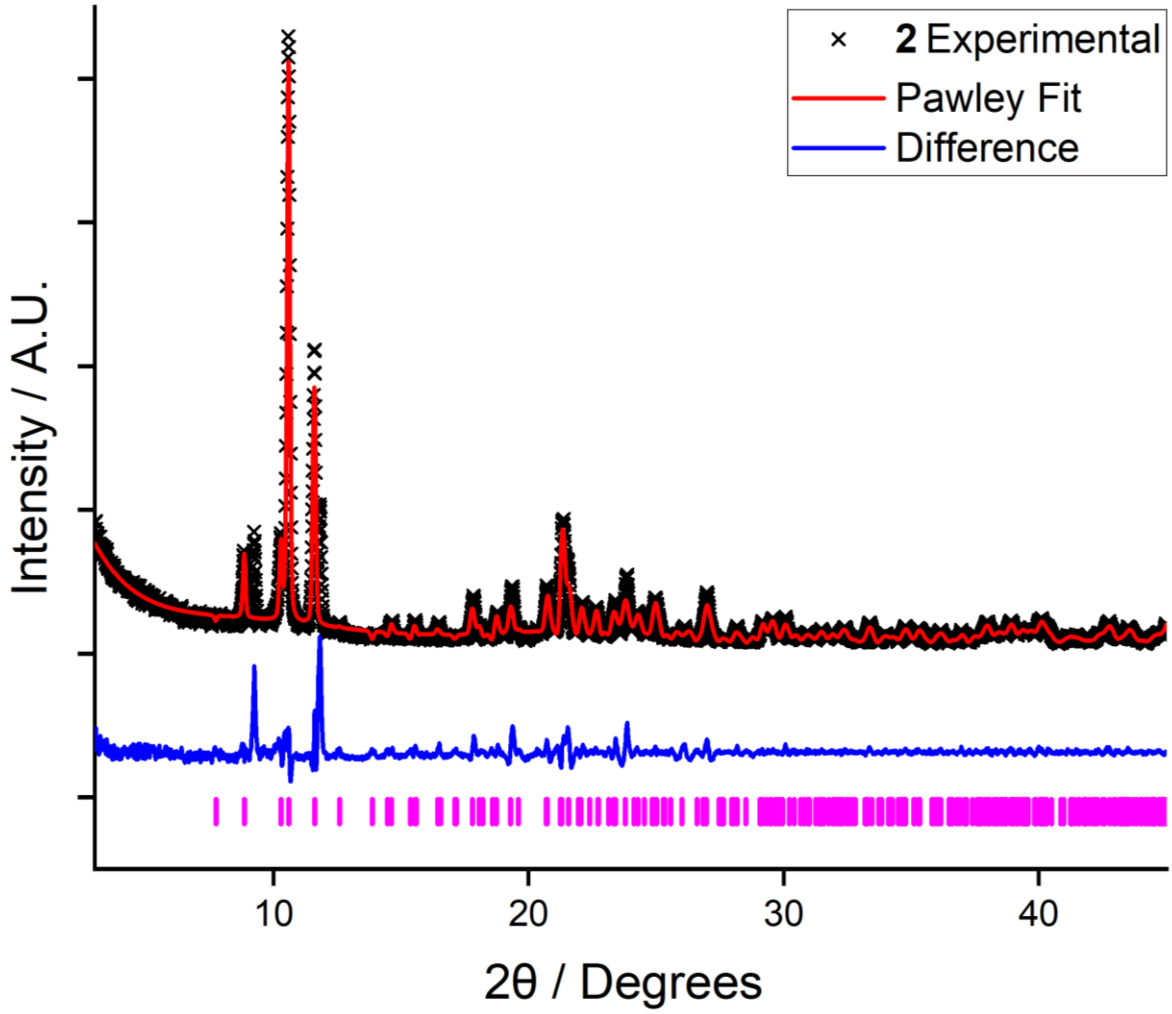

Figure S21. Pawley fit of the bulk experimental pattern of **2** (as synthesised). Pawley fit yields orthorhombic unit cell parameters; a = 12.236(2) Å, b = 15.233(2) Å, c = 17.153(4) Å, V = 3197(1) Å^3 (R_{wp} = 16.8%).

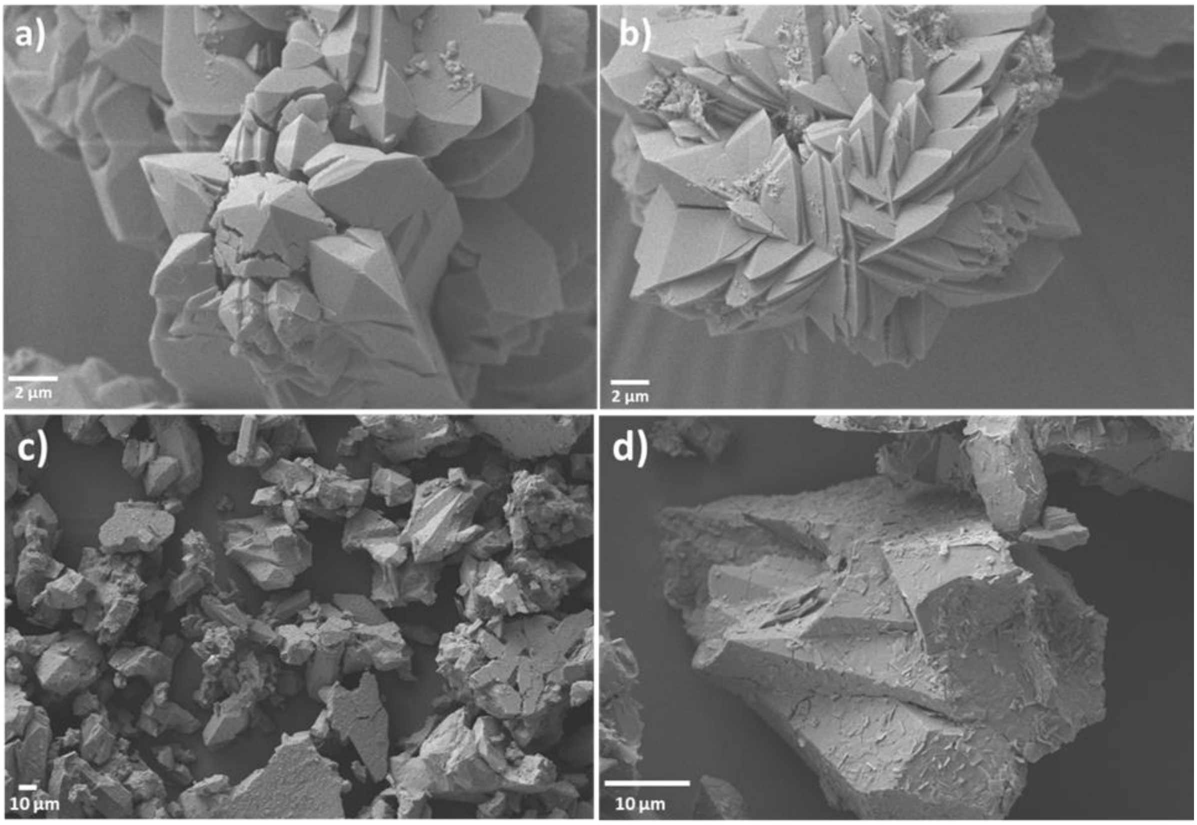

Figure S22. SEM images of **2** dried from (a/b) *N*,*N*-dimethylformamide and (c/d) acetonitrile.

The ^{1}H NMR spectrum of an acid-digested sample of **2** confirms the presence of formate bridges in the framework (Figure S23). The formate proton resonates at δ = 8.1 ppm, in an integral ratio of 1.1:6 with the two equivalent protons of the fumarate linker (δ = 6.6 ppm). This supports the results reported using single crystal X-ray diffraction data, wherein there is one formate bridge to three ligands. DMF is also present in a 1:3 molar ratio with the fumarate ligands, aligned to the crystallographic data.

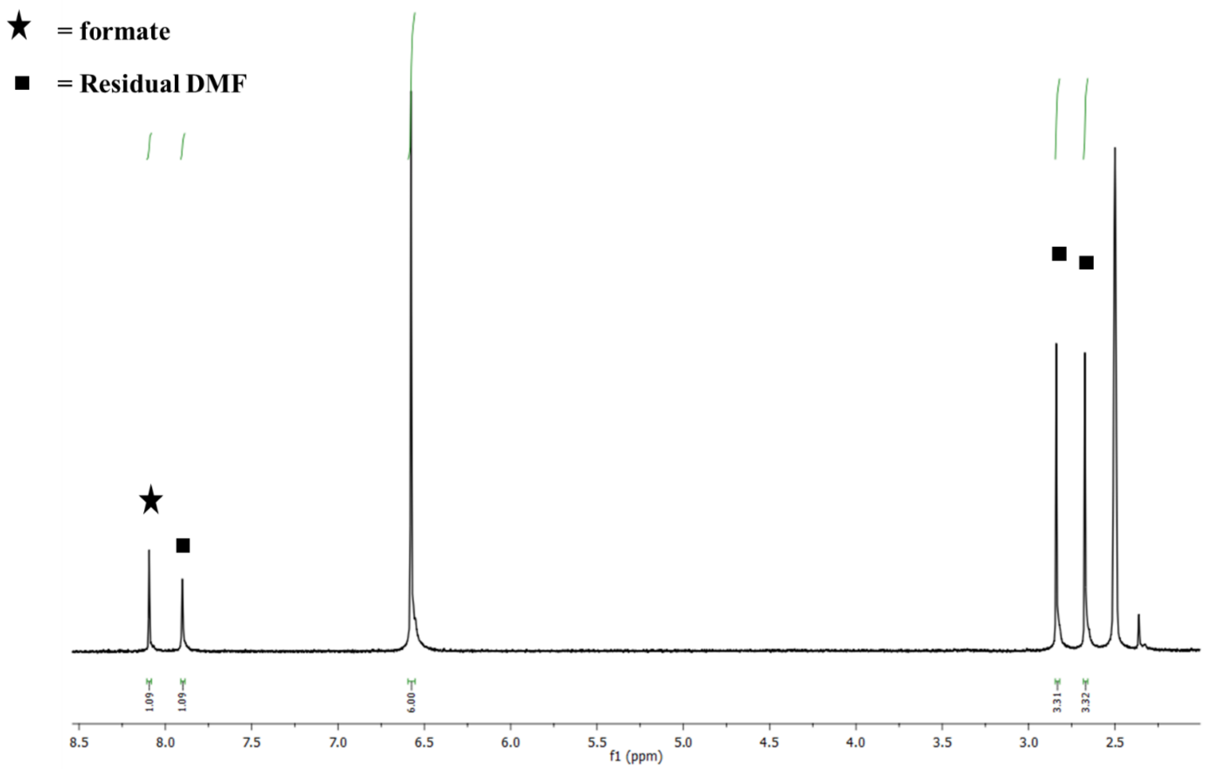

Figure S23. Partial ^{1}H NMR spectrum (DMSO-d_6 / D_2SO_4) of **2**. δ/ppm: 2.67 (s, 3.3H), 2.84 (s, 3.3H), 6.58 (s, 6H), 7.9 (s, 1.1H), 8.09 (s, 1.1H).

The thermal stability of **2** was analysed using thermogravimetric analysis in air (Figure S24). The sample was heated to 800 °C at a rate of 10 °C min^{-1} and exhibits a clear one-step mass loss. This event is coincident with the loss of ligand and subsequent breakdown of the MOF at 450 °C. The resultant residual mass is 31.8% wt, which scales to 32.4% wt when weakly adsorbing residues below 100 °C are not considered. This value is accordant with the expected residual mass for Sc_2O_3 (33.4% wt) calculated from the formula $[Sc_3O(C_4H_2O_4)_3(HCO_2)(H_2O)_{0.5}(C_3H_7NO)_{0.5}]\cdot(C_3H_7NO)_{0.5}$.

Elemental analysis suggested a slightly higher amount of DMF in the particular sample of **2** analysed, with the nitrogen and carbon contents suggesting an additional 0.4 molecules of DMF per Sc_3O cluster in this particular sample. Elemental analysis calculated for $[Sc_3O(C_4H_2O_4)_3(HCO_2)(H_2O)_{0.5}(C_3H_7NO)_{0.5}]\cdot(C_3H_7NO)_{0.9}$: C, 31.81%; H, 2.76%; N, 3.02%. Found: C, 32.22%; H, 2.96%; N, 2.70%.

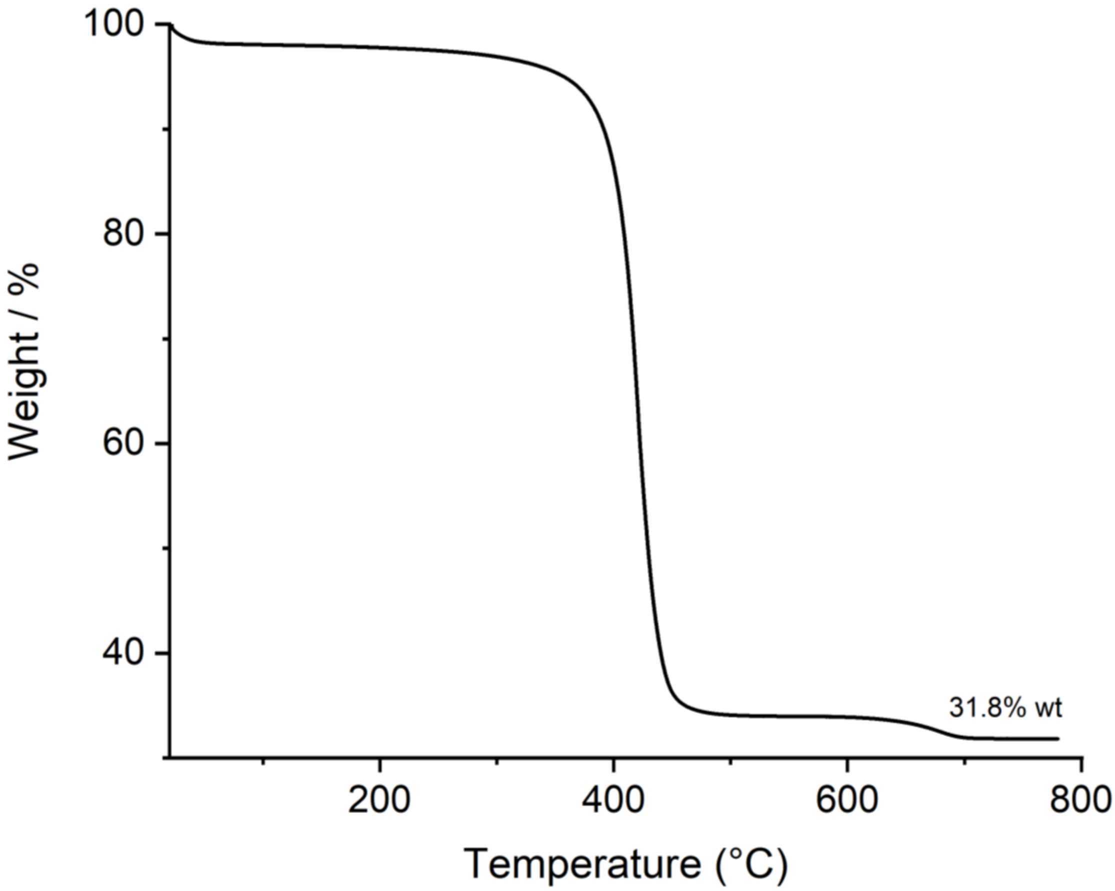

Figure S24. TGA profile of **2**, yielding a residual mass of 31.8% wt. When scaled to remove weakly adsorbing residues that are lost below 100 °C, the residual mass is 32.4% wt.

In order to understand the porosity of **2**, nitrogen, carbon dioxide and hydrogen sorption experiments were carried out. **2** exhibits permanent porosity and a type I isotherm for N_2 adsorption at 77 K; the BET surface area of the framework was found to be 923 m^2 g^{-1} (Figure S25). The framework also shows reasonable hydrogen storage capacity, reaching almost 1.4 wt% at 1 bar. This, coupled with the good CO_2 uptake at 1 bar at 273 K highlights the potential for this MOF to be further investigated for gas capture.

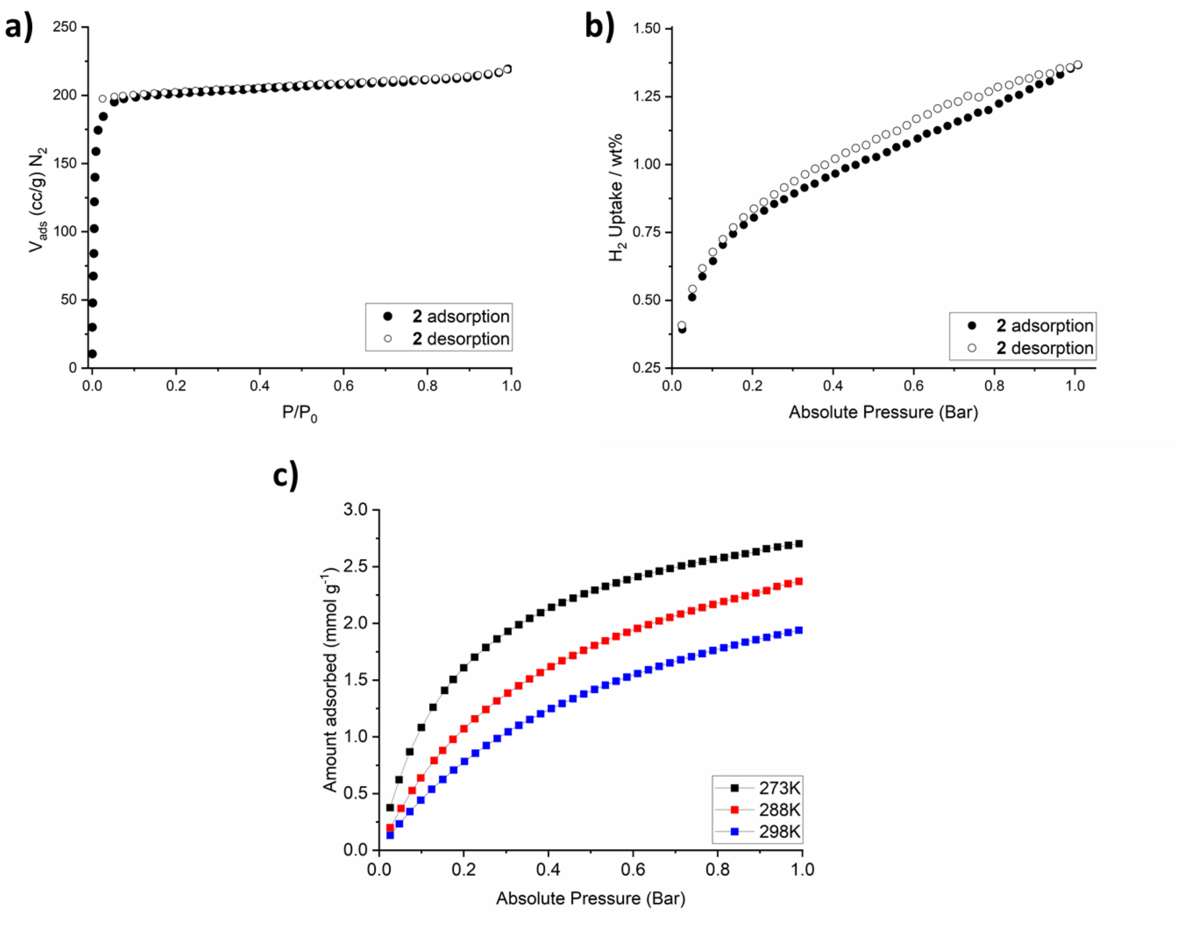

Figure S25. a) N_2 adsorption/desorption isotherm for **2**, collected at 77 K. b) H_2 adsorption/desorption isotherm for **2**, collected at 77 K. c) CO_2 adsorption isotherms of **2**, at varying temperatures collected at 298 K (blue), 283 K (red) and 273 K (black).

S7. Bromination of 1

Single Crystal Solution Bromination of 1*as*: In a 20 mL scintillation vial, crystals of **1*as*** were soaked in acetonitrile solution for 1 hour. Fresh MeCN (2 mL) was exchanged using a Pasteur pipette. Bromine (10 µL) was added, the vial was sealed, covered in tin foil and left to stand for 48 hours. Following this, the MeCN was replaced several times with fresh solvent and the crystals were stored in MeCN until further analysis.

Crystal data for 1-Br$_2$: $Sc_3O(C_4H_2O_4Br_2)_3(H_2O)_2(OH)$, M_r = 1025.55, crystal dimensions 0.1 x 0.06 x 0.02 mm, Hexagonal; *a* = *b* = 13.3462 (14) Å, *c* = 13.5382 (12) Å, *V* = 2088.34(5) Å^3, *T* = 150 K, space group *P*6$_3$/*m* (No. 176), *Z* = 2, 14696 reflections measured, 1338 independent reflections (R_{int} = 0.149) which were all used in calculations. The final R_1 = 0.091 for 902 observed data $R[F^2 > 2\sigma(F^2)]$ and $wR(F^2)$ = 0.288 (all data). CCDC deposition number 2150611.

Bulk Phase Vapour Bromination of 1*np*: Bulk powder of **1*np*** (0.04 g, 0.220 mmol alkene, 1 eq) was added to a small scintillation vial and liquid bromine (170 µL, 3.30 mmol, 15 eq) was added to a separate scintillation vial. The two vials were sealed together in a larger scintillation vial, capped and stored in the dark for 48 hours. The product, **1-Br$_2$**, was washed with acetonitrile until the supernatant was clear, before being placed in the desiccator under vacuum at room temperature to dry prior to analysis.

Following bromination, the purity of the MOF, **1-Br$_2$**, was determined using PXRD, both by comparison with the pattern predicted from the single crystal structure of **1-Br$_2$** (Figure S26) and through Pawley refinement (Figure S27).

When as-synthesised **1-Br$_2$** was dried under vacuum (20 h at 150 °C) and subject to gas adsorption analysis, the crystallinity and unit cell did not change. This indicates that, unlike **1**, the framework is now rigid (Figure S26). Pawley fitting of **1-Br$_2$** yields a hexagonal unit cell with parameters *a* = *b* = 13.499(13) Å, *c* = 13.489(3) Å (R_{wp} = 8.59%, Figure S27). This is a close fit with the predicted structure which has unit cell values of *a* = *b* = 13.3462(14) Å, *c* = 13.5382(12) Å.

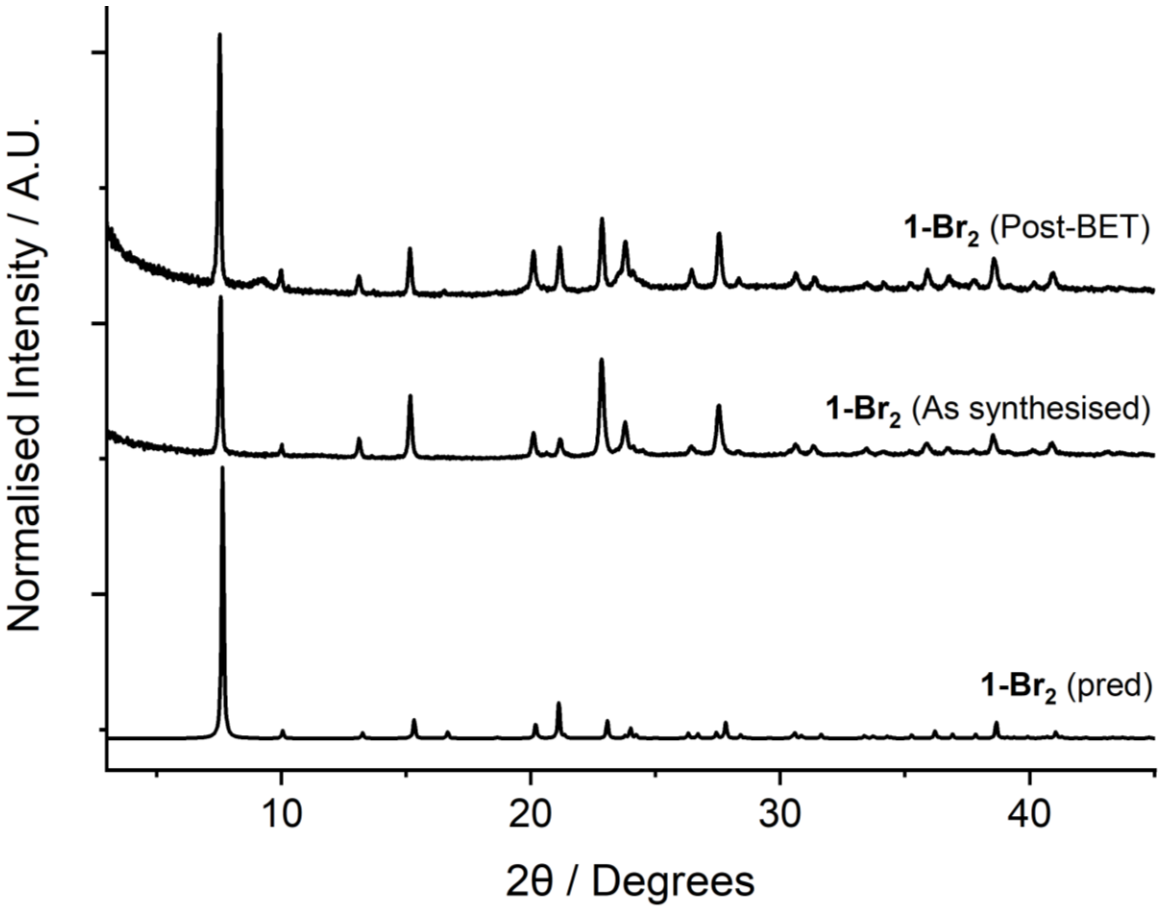

Figure S26. Stacked PXRD pattern of bulk samples of **1-Br**$_2$ (as synthesised) and **1-Br**$_2$ (post-BET) compared to the predicted PXRD pattern from the crystal structure of **1-Br**$_2$.

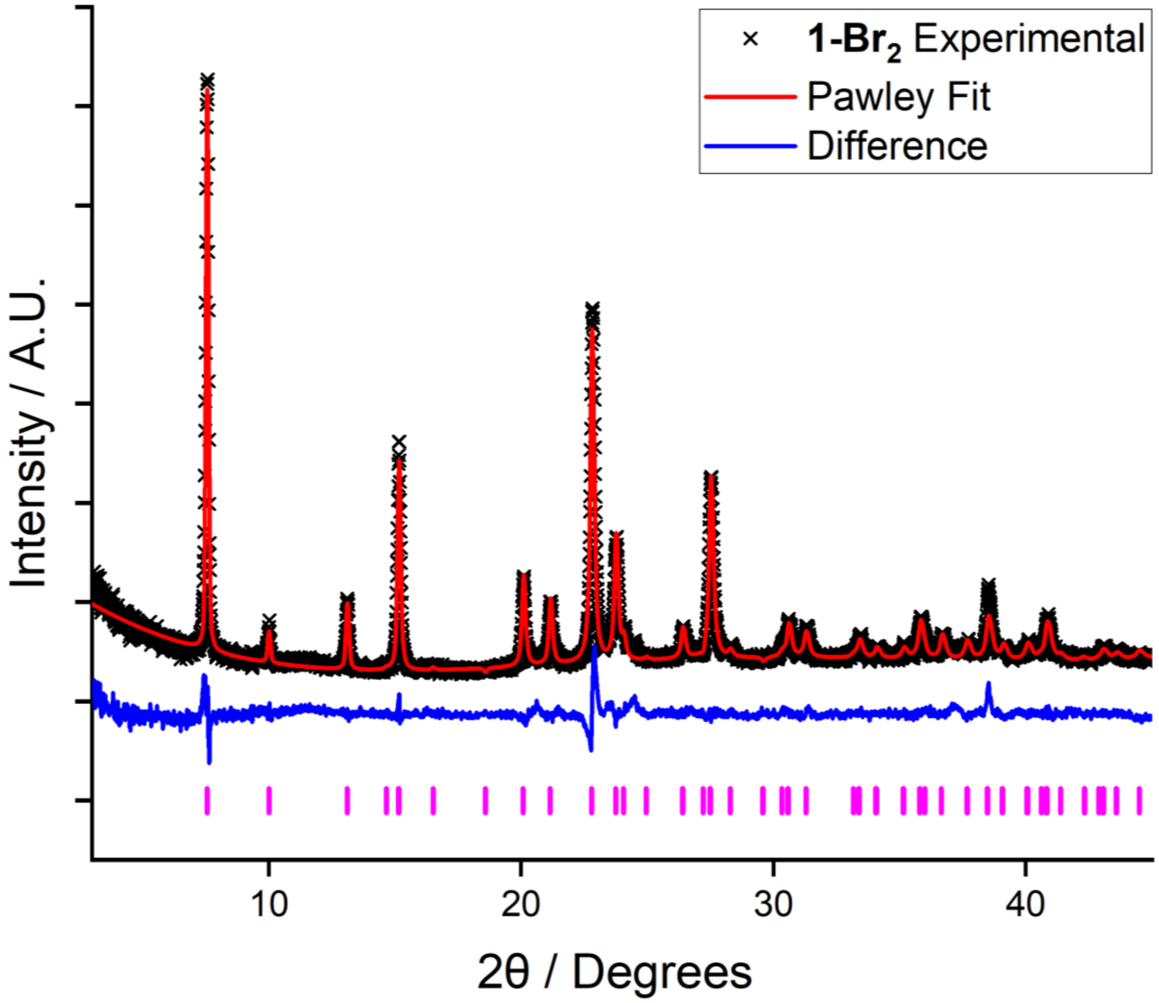

Figure S27. Pawley fit of the bulk experimental pattern of **1-Br$_2$** (as synthesised). Pawley fit yields hexagonal unit cell parameters; $a = b = 13.499(13)$ Å, $c = 13.489(3)$ Å, $V = 2129(2)$ Å^3 ($R_{wp} = 8.59\%$).

To further confirm the rigidity, **1-Br$_2$** was soaked in DMF for 24 hours and subsequently analysed using PXRD (Figure S28). Following exposure to air overnight on the diffractometer, there is no apparent change in PXRD pattern, confirming that the framework shows no tendency to flex.

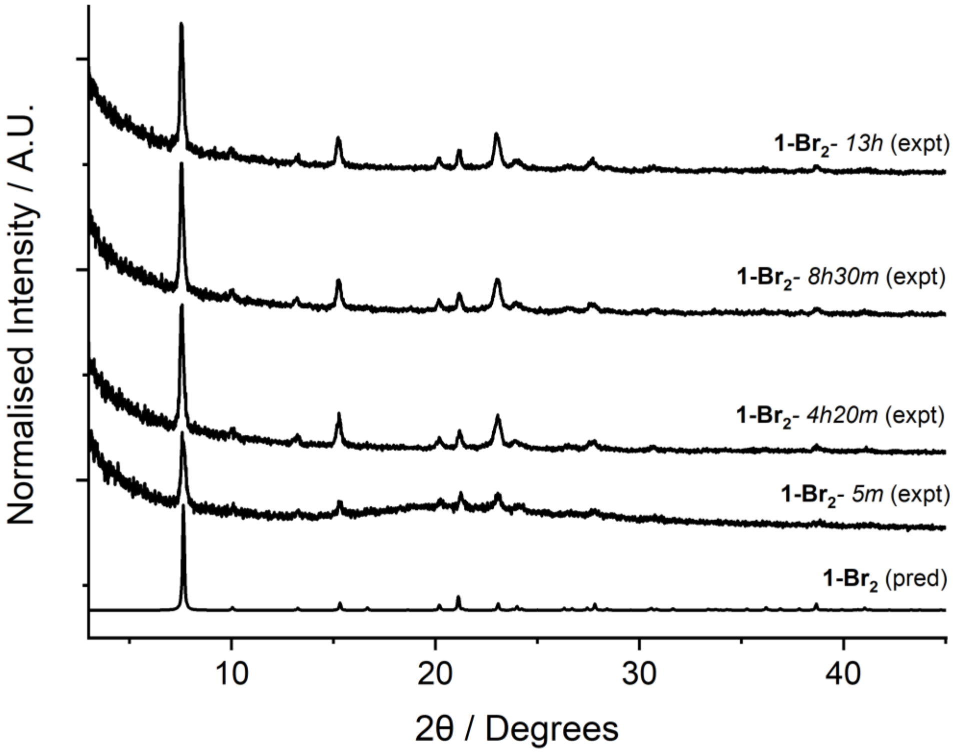

Figure S28. PXRD patterns of **1-Br$_2$** in DMF, collected over 13 hours during in-situ exposure to air (h = hours).

Further confirmation of the conversion from **1** to **1-Br$_2$** is evident by using NMR spectroscopy. In the ^{1}H NMR spectrum of an acid-digested sample (Figure S29), the peak representing alkene C-H protons (δ = 6.5 ppm) disappears upon bromination, while a resonance appears at δ = 4.5 ppm that is assigned to the bromoalkane protons of the modified linker. This is supported by the disappearance of a resonance at δ = 134 ppm, assigned to the *sp*^{2} alkenyl C atoms, in the ^{13}C NMR spectrum, and the emergence of the resonance assigned to the *sp*^{3} carbons at δ = 45 ppm (Figure S30). This confirms the quantitative conversion of **1** to **1-Br$_2$**. The ^{1}H NMR spectrum also showed the retention of DMF, with integrals showing the same approximately 1.5 moles of DMF per Sc_3O cluster ratio in **1-Br$_2$** that is observed in the parent MOF **1*np***.

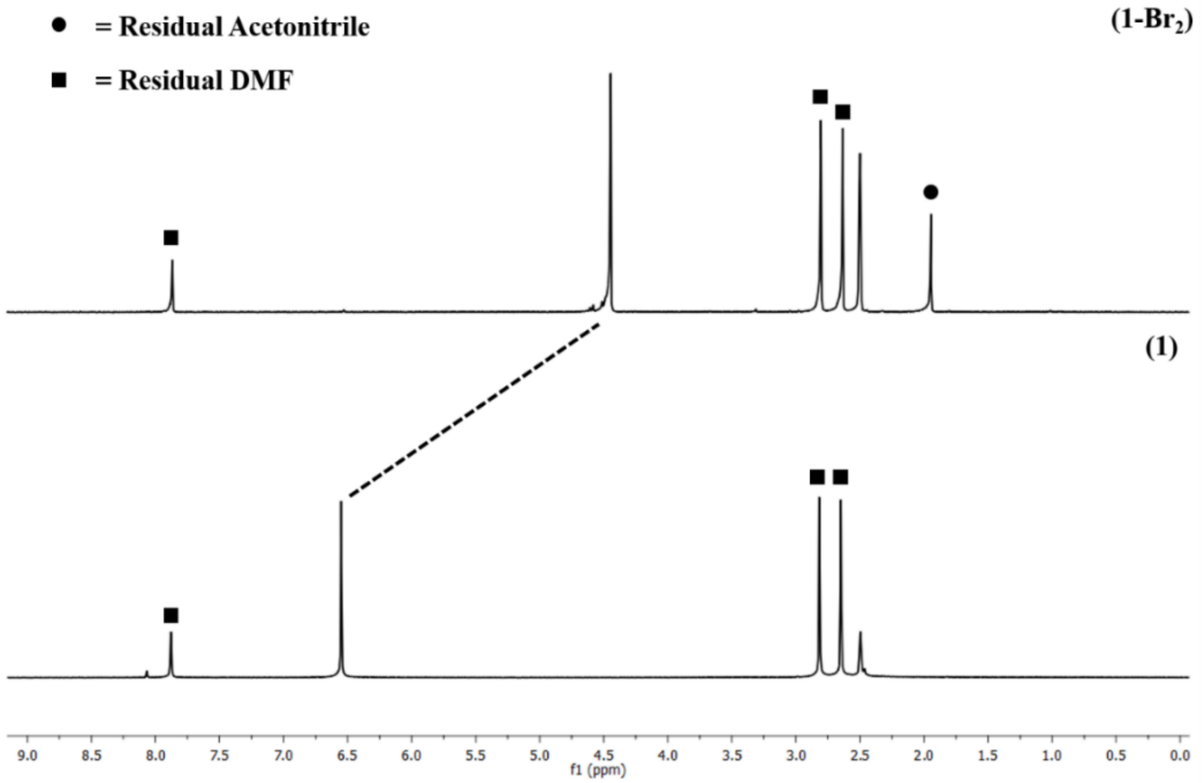

Figure S29. Stacked ^{1}H NMR spectra of the conversion of **1** to **1-Br**$_2$ following digestion in DMSO-d_6/D_2SO_4. **1-Br**$_2$ δ/ppm: 2.64 (s, 4.9H), 2.80 (s, 4.9H), 4.45 (s, 6H), 7.87 (s, 1.4H). This gives a linker to DMF ratio of 3:1.63, or a Sc_3O cluster to DMF ratio of 1:1.63.

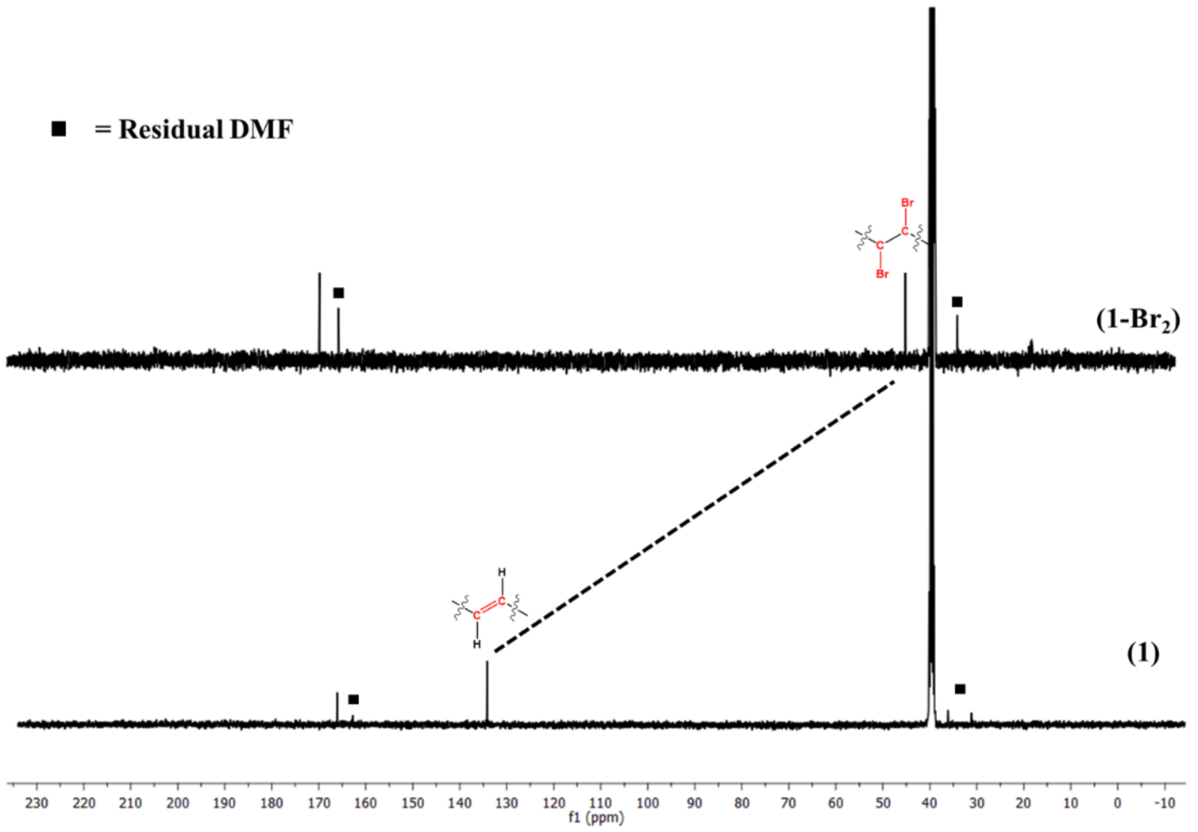

Figure S30. Stacked ^{13}C NMR spectra of the conversion of **1** to **1-Br**$_2$ following digestion in DMSO-d_6/D_2SO_4.

Raman spectroscopy is also successfully used to monitor this chemical transformation (Figure S31). The alkene moiety is represented by the signal at 1670 cm^{-1}. Following bromination, this signal is lost and a new signal at 641 cm^{-1} appears. The latter is indicative of the C-Br stretch.

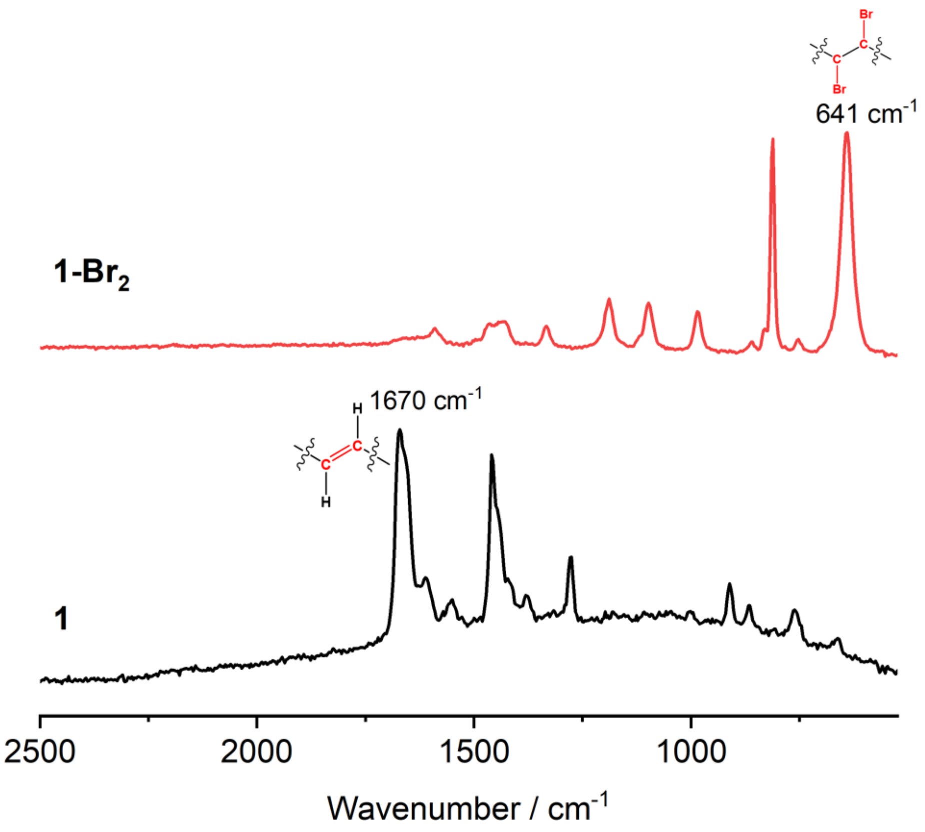

Figure S31. Stacked Raman spectra to highlight the transformation from **1** (black) to **1-Br**$_2$ (red).

Thermogravimetric analysis was also used to understand the change in thermal stability between **1** and **1-Br**$_2$ (Figure S32). The resultant residual mass is 16.8% wt which, when scaled to remove any weakly absorbing residues below 100°C, becomes 17.5% wt and is accordant with the expected residual mass of Sc_2O_3 (18.2% wt) calculated from the formula $[Sc_3O(C_4H_2O_4Br_2)_3(H_2O)_2(OH)(C_3H_7NO)_{1.5}]$. Unlike **1**, which only had one distinct mass loss step accounting for the linker, **1-Br**$_2$ shows multiple overlapping steps in the TGA profiles, likely a result of cleavage of C-Br bonds and the subsequent loss of bromine (Figure S33).

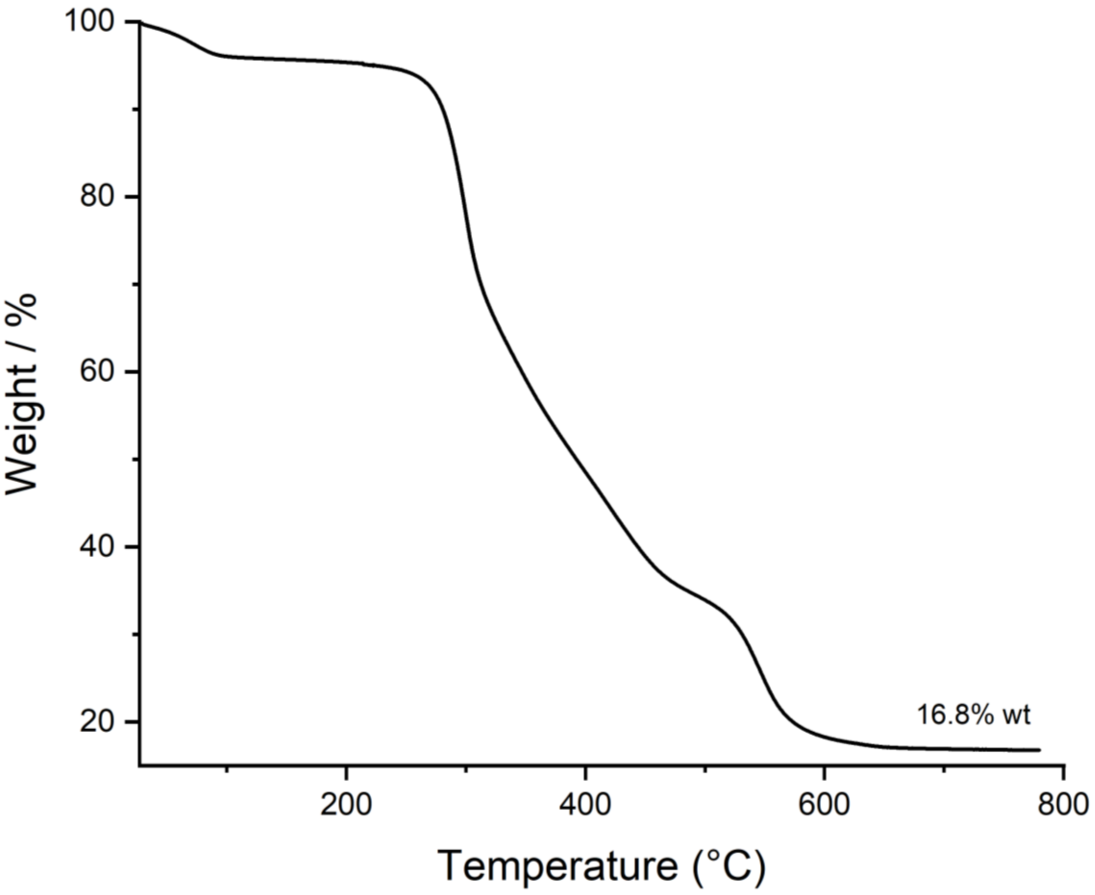

Figure S32. TGA profile of **1-Br$_2$** after activation and gas adsorption analysis, yielding a residual mass of 16.8% wt. When scaled to remove weakly adsorbing residues that are lost below 100 °C, the residual mass is 17.5% wt.

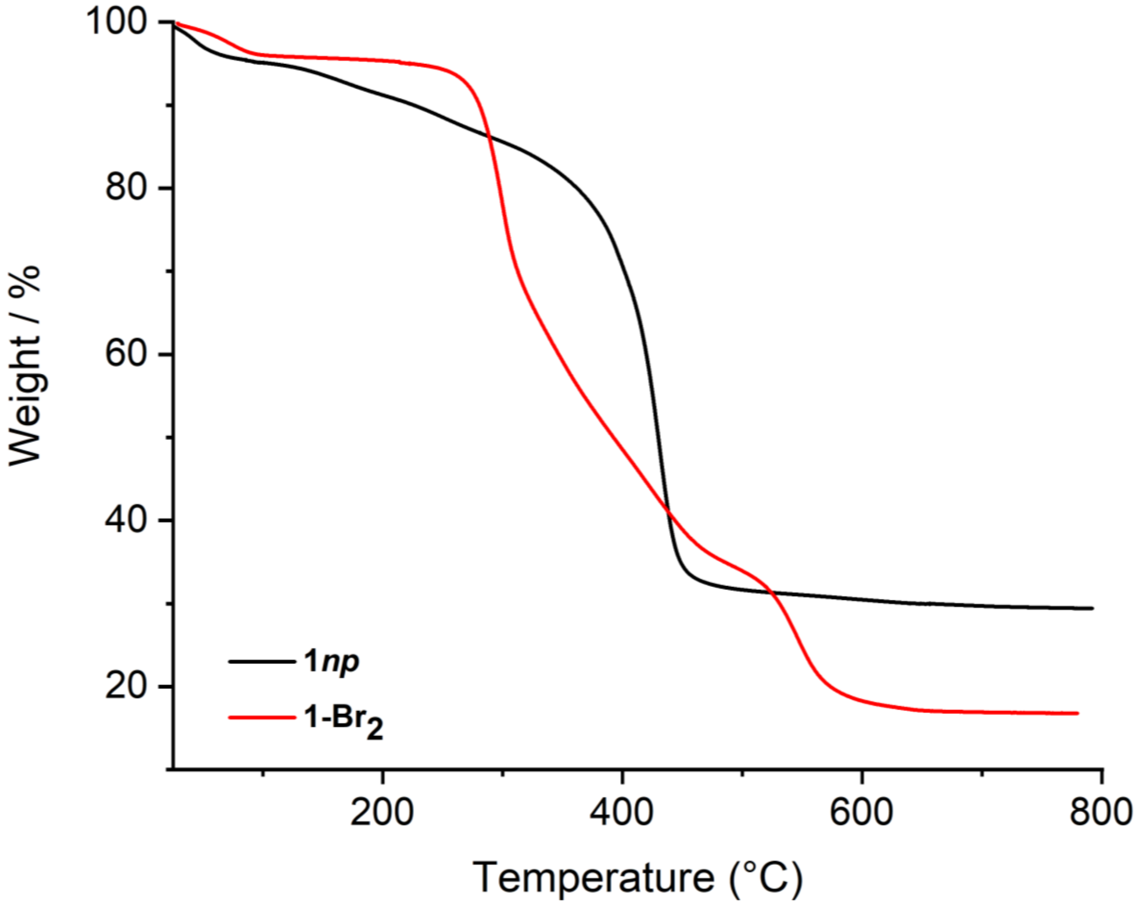

Figure S33. TGA profile of **1*np*** vs **1-Br$_2$** illustrating the 2-step mass loss observed in **1-Br$_2$**.

Elemental analysis, however, gave C and N contents lower than would be expected, correlating more closely with only one DMF molecule per SBU and adsorption of one water molecule, suggesting a formula of $[Sc_3O(C_4H_2O_4Br_2)_3(H_2O)_2(OH)]\cdot C_3H_7NO\cdot H_2O$ for this particular sample; the theoretical bromine content from this composition (42.9% wt) correlates well with the experimentally measured value of 45.7% wt. Elemental analysis calculated for $[Sc_3O(C_4H_2O_4Br_2)_3(H_2O)_2(OH)]\cdot(C_3H_7NO)\cdot(H_2O)$: C, 16.14%; H, 1.81%; N, 1.25%; Br, 42.94%. Found: C, 15.94%; H, 1.98%; N, 1.39%; Br, 45.70%.

S8. Bromination of 2

Bulk Phase Vapour Bromination of 2: 2 was washed three times in acetonitrile and dried overnight in a desiccator under vacuum at room temperature. Bulk powder of **2** (0.03 g, 0.137 mmol alkene, 1 eq) was added to a small scintillation vial and liquid bromine (70 µL, 1.371 mmol, 10 eq) was added to a separate scintillation vial. The two vials were sealed together in a larger scintillation vial, capped and stored in the dark for 48 hours. The product, **2-Br$_2$**, was washed with acetonitrile until the supernatant was clear, before being placed in the desiccator under vacuum at room temperature to dry prior to analysis.

The transformation from **2** to **2-Br$_2$** was monitored using powder X-ray diffraction (Figure S34). Using this method, it is clear that the rigid orthorhombic framework, **2**, has been converted back to the previously discussed hexagonal **1-Br$_2$** framework.

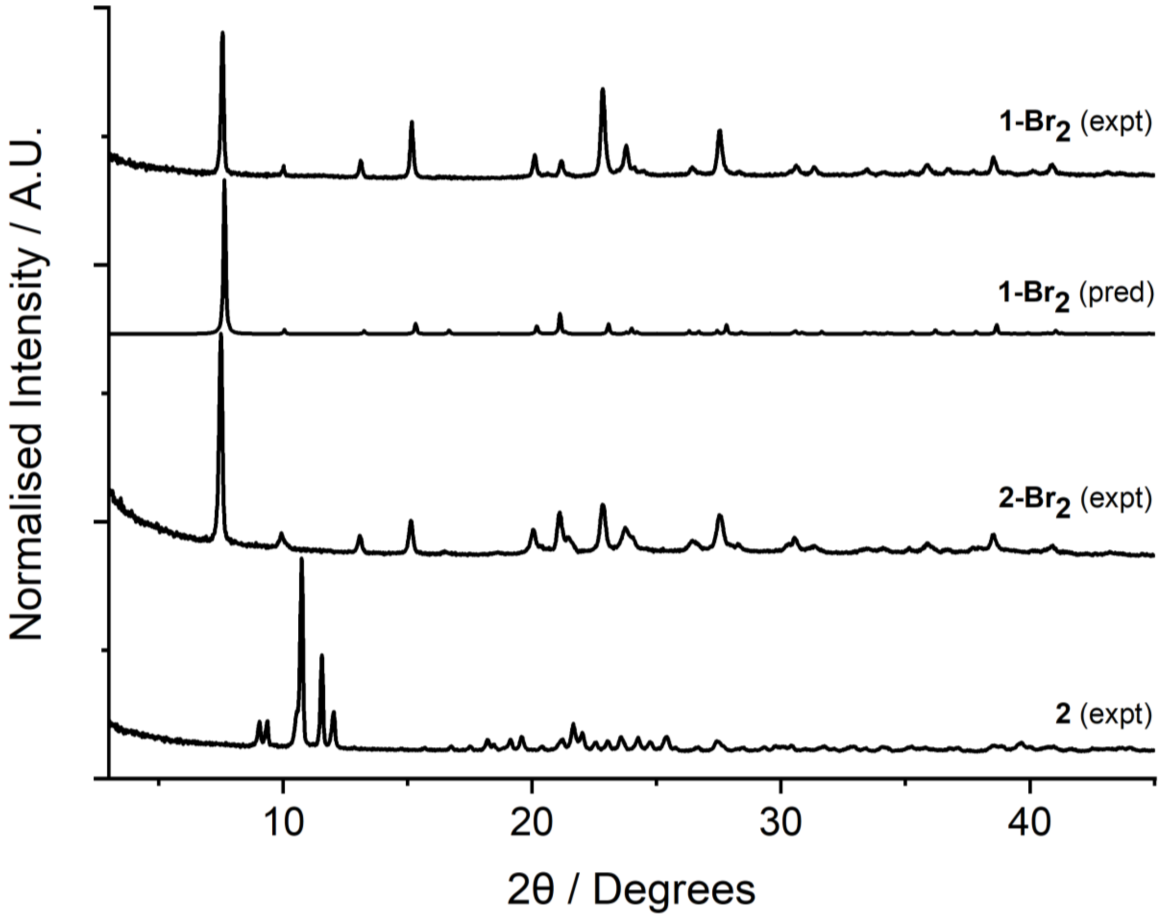

Figure S34. Stacked PXRD patterns of the chemical transformation from **2** to **2-Br$_2$**, compared to predicted and experimental patterns for **1-Br$_2$**.

^{1}H NMR spectroscopy has been used to confirm the quantitative conversion from **2** to **2-Br$_2$**. As reported with **1-Br$_2$**, the C-H alkene resonance at δ = 6.6 ppm disappears and an alkane C-H peak emerges at δ = 4.6 ppm, indicative of the conversion from *sp*^{2} to *sp*^{3} centres as a direct result of bromination. Noticeably, the peak representing the formate bridge at δ = 8.1 ppm disappears following bromination (Figure S35). This indicates the susceptibility of the bridge to attack and results in the rigid, porous **2** converting to the same product as **1-Br$_2$**; presumably the formate is removed during the washing step. The bromination is also evident in the ^{13}C NMR spectra, whereby the peak at δ = 134 ppm representing *sp*^{2} carbon centres disappears and is followed by the emergence of a resonance assigned to the C-Br *sp*^{3} carbon at δ = 43 ppm (Figure S36).

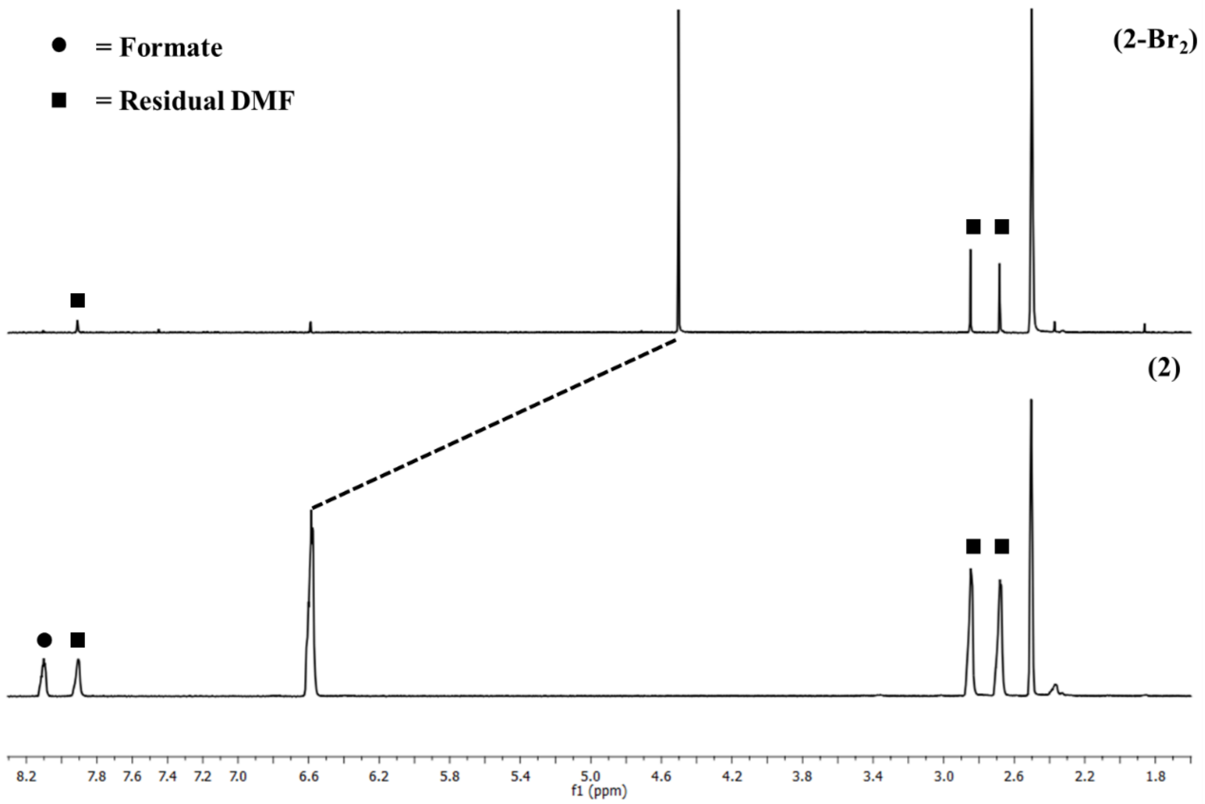

Figure S35. Stacked ^{1}H NMR spectra (DMSO-*d*$_6$/D_2SO_4) showing the conversion from **2** to **2-Br$_2$**. **2-Br$_2$** δ/ppm: 2.68 (s, 2.4H), 2.85 (s, 2.4H), 4.50 (s, 6H), 7.91 (s, 0.76H). This gives a linker to DMF ratio of 3:0.8, or a Sc_3O cluster to DMF ratio of 1:0.8, which corresponds well to the 0.75 moles of DMF predicted by elemental analysis.

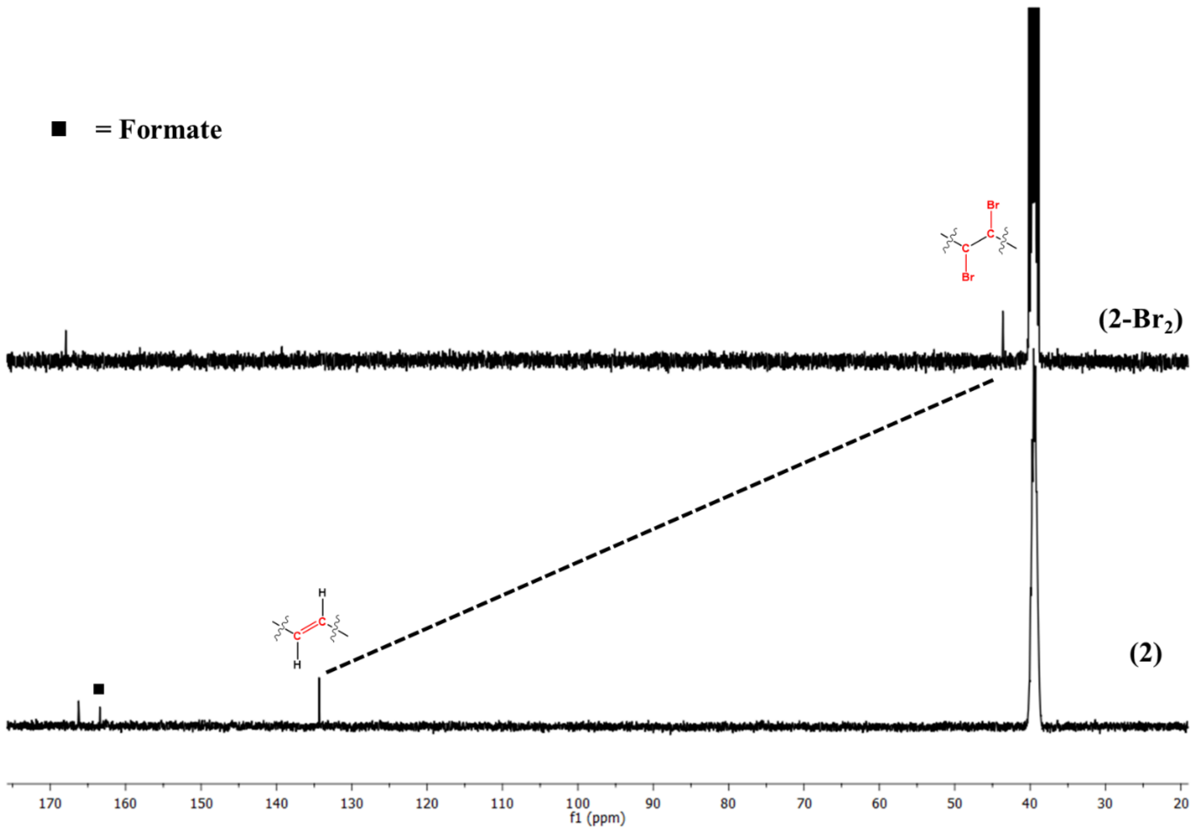

Figure S36. Stacked ^{13}C NMR spectra (DMSO-*d*$_6$/D_2SO_4) showing the conversion from **2** to **2-Br$_2$**.

Raman spectroscopy is also successfully used to monitor this chemical transformation (Figure S37). As seen in the transformation of **1** to **1-Br$_2$**, following bromination of **2**, the alkene signal at 1670 cm^{-1} disappears and a new signal at 635 cm^{-1} appears. The latter is indicative of the C-Br stretch in **2-Br$_2$**.

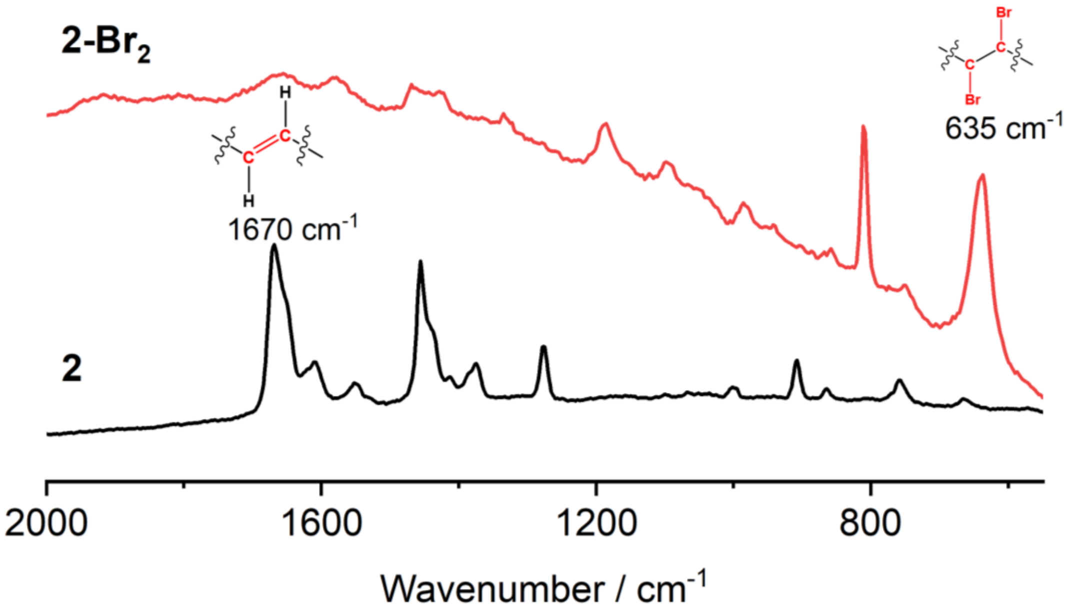

Figure S37. Stacked Raman spectra to highlight the transformation from **2** (black) to **2-Br**$_2$ (red).

Thermogravimetric analysis was used to determine the thermal stability of **2-Br**$_2$ and the resultant residue of the framework, following heating to 800 °C (Figure S38). Assuming the initial mass loss below 100 °C is solvent within the pores, a large mass loss occurs at approximately 300 °C and can be assigned to the loss of brominated fumarate linkers ($C_4H_2O_4Br_2$). The residual mass is 18% wt which, when scaled to remove any weakly absorbing residues below 100°C gives a value of 19.1% wt, agreeing very closely with the expected residual mass of Sc_2O_3 (19.1% wt) based on the previously proposed formula; $[Sc_3O(C_4H_2O_4Br_2)_3(H_2O)_2(OH)]\cdot(C_3H_7NO)_{0.75}$.

As observed for **1-Br**$_2$, **2-Br**$_2$ exhibits multiple mass loss steps in the TGA profile compared to the one distinct mass loss exhibited by **2** (Figure S39).

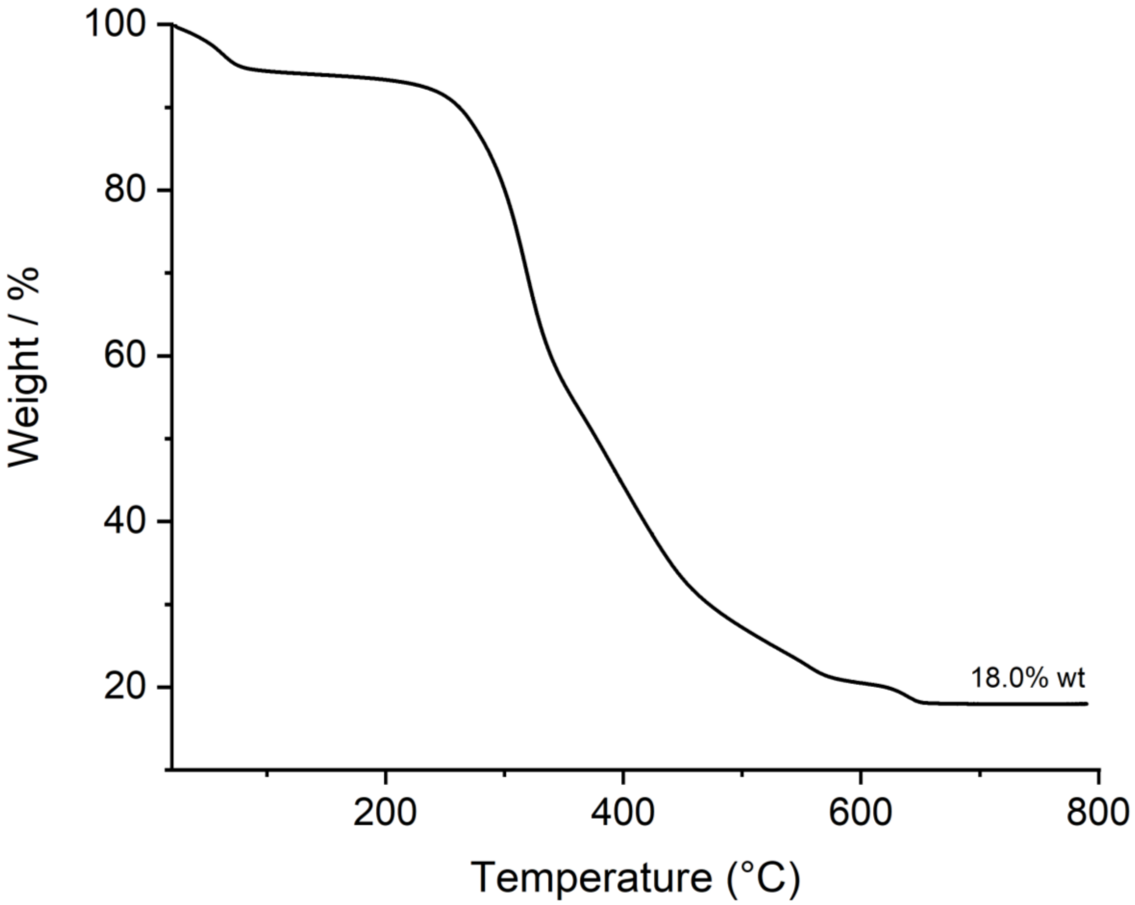

Figure S38. TGA profile for **2-Br$_2$**, after activation and gas adsorption analysis, yielding a residual mass of 18.0% wt. When scaled to remove weakly adsorbing residues that are lost below 100 °C, the residual mass is 19.1% wt.

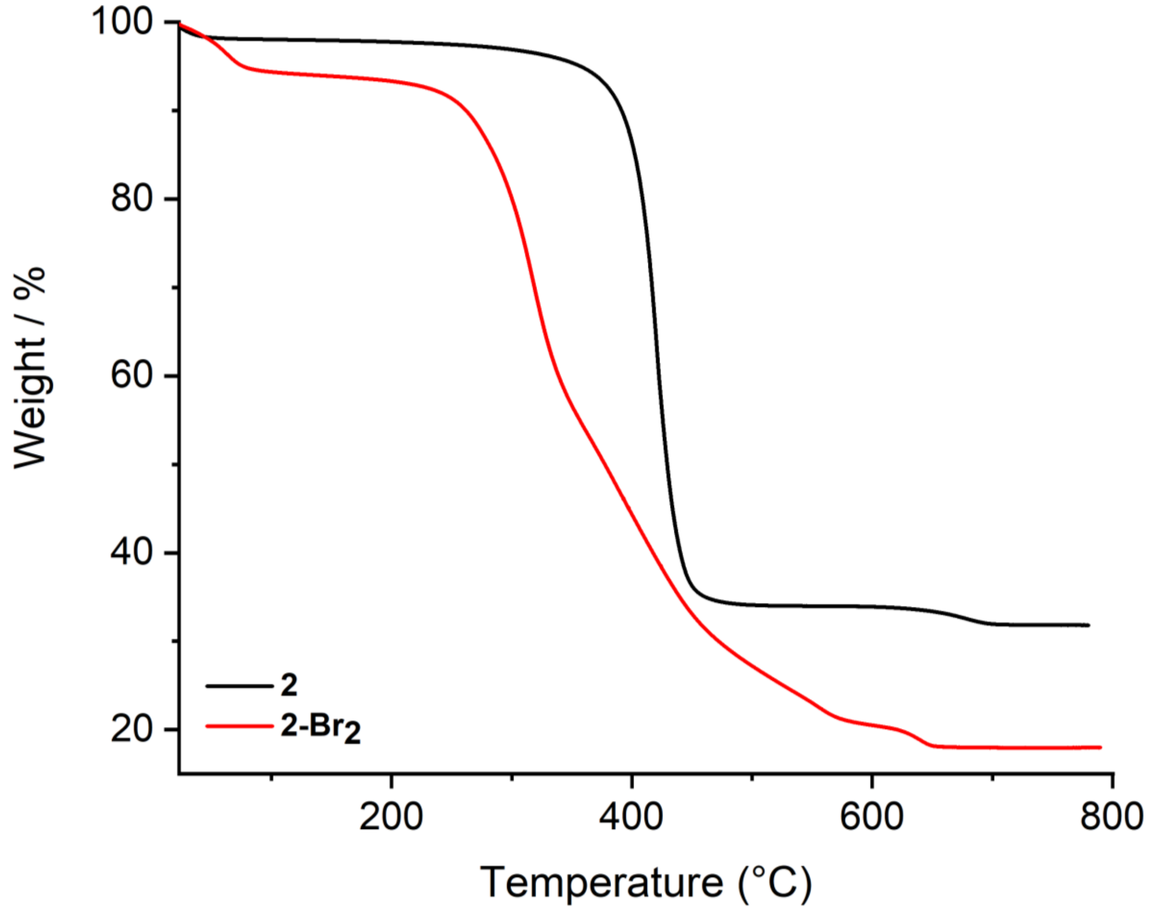

Figure S39. TGA profiles of **2** vs **2-Br₂** illustrating the multiple mass loss steps observed in **2-Br₂**.

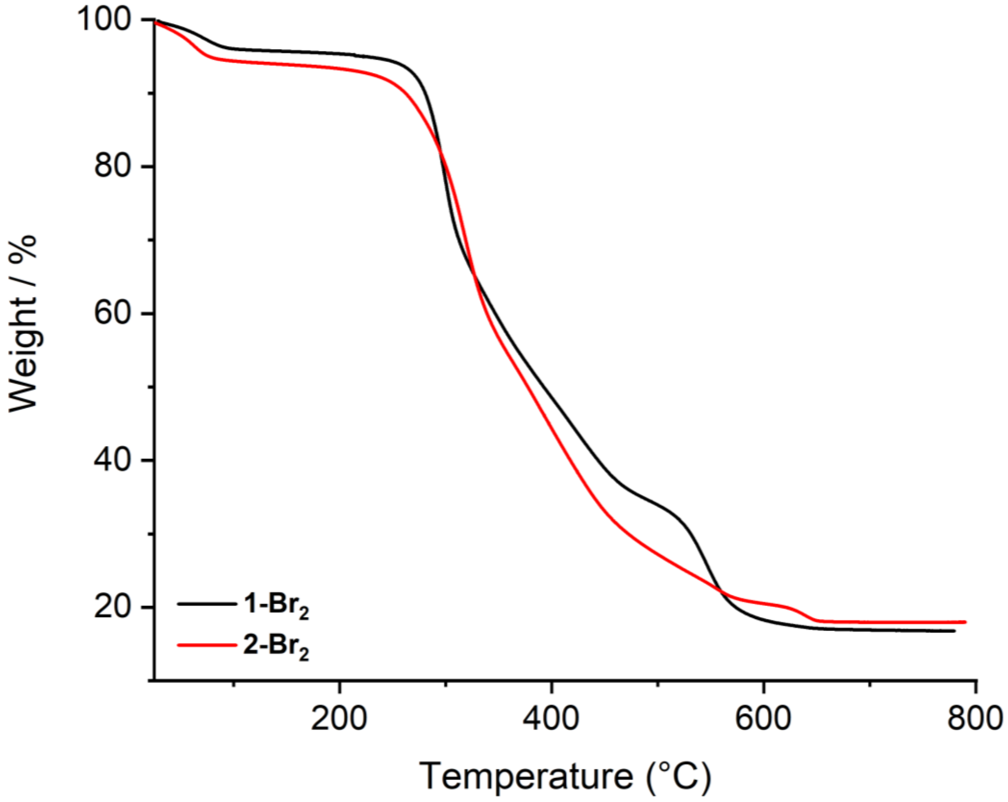

Figure S40. TGA profiles of **1-Br$_2$** vs **2-Br$_2$** illustrating the similarities in their profiles when heated in air to 800 °C.

Elemental analysis was also confirmed bromination and retention of DMF, but this particular sample seemingly adsorbed atmospheric water (2 moles per mole of Sc_3O cluster) prior to analysis. Nevertheless, it confirms that bromination occurs at the linker sites and that the bridging formate is not replaced by additional bromide counterions. Elemental analysis calculated for $[Sc_3O(C_4H_2O_4Br_2)_3(H_2O)_2(OH)]\cdot(C_3H_7NO)_{0.75}\cdot(H_2O)_2$: C, 15.33%; H, 1.83%; N, 0.94%; Br, 42.95%. Found: C, 14.90%; H, 2.25%; N, 1.16%; Br, 43.51%.

S9. References

[S1] B. H. Toby, R. B. Von Dreele, *J. Appl. Crystallogr.* **2013**, *46*, 544-549.

[S2] G. M. Sheldrick, *Acta Cryst. A* **2015**, *71*, 3-8.

[S3] G. M. Sheldrick, *Acta Cryst. C* **2015**, *71*, 3-8.

[S4] O. V. Dolomanov, L. J. Bourhis, R. J. Gildea, J. A. K. Howard, H. Puschmann, *J. Appl. Crystallogr.* **2009**, *42*, 339-341.

[S5] A. L. Spek, *Acta Cryst. C* **2015**, *71*, 9-18.

[S6] A. L. Spek, *J. Appl. Crystallogr.* **2003**, *36*, 7-13.

[S7] C. Mellot-Draznieks, C. Serre, S. Surblé, N. Audebrand, G. Férey, *J. Am. Chem. Soc.* **2005**, *127*, 16273-16278.

[S8] C. Serre, C. Mellot-Draznieks, S. Surblé, N. Audebrand, Y. Filinchuk, G. Férey, *Science* **2007**, *315*, 1828-1831.

[S9] a) J. VandeVondele, M. Krack, F. Mohamed, M. Parrinello, T. Chassaing, J. Hutter, *Comput. Phys. Commun.* **2005**, *167*, 103-128; b) J. Hutter, M. Iannuzzi, F. Schiffmann, J. VandeVondele, *Wiley Interdiscip. Rev. Computat. Mol. Sci.* **2014**, *4*, 15-25.

[S10] J. VandeVondele, J. Hutter, *J. Chem. Phys.* **2007**, *127*, 114101-114109.

[S11] a) S. Goedecker, M. Teter, J. Hutter, *Phys. Rev. B* **1996**, *54*, 1703-1710; b) M. Krack, *Theor. Chem. Acc.* **2005**, *114*, 145-152.

[S12] J. P. Perdew, K. Burke, M. Ernzehof, *Phys. Rev. Lett.* **1996**, *77*, 3865-3868.

[S13] S. Grimme, J. Antony, S. Ehrlich, H. Krieg, *J. Chem. Phys.* **2010**, *132*, 154104-154119.
